# Emotion regulation in prolonged grief disorder in later life: Protocol and rationale for a longitudinal neuroimaging study

**DOI:** 10.1162/IMAG.a.1271

**Published:** 2026-06-16

**Authors:** Gyujoon Hwang, Nutta-on P. Blair, Volkan Emre Arpinar, B. Douglas Ward, Timothy L. McAuliffe, Stacy A. Claesges, Andrew S. Nencka, Yang Wang, Charles F. Reynolds, Elliot A. Stein, Joseph S. Goveas

**Affiliations:** Department of Psychiatry and Behavioral Medicine, Medical College of Wisconsin, Milwaukee, WI, United States; Department of Radiology, Medical College of Wisconsin, Milwaukee, WI, United States; Department of Psychiatry, University of Pittsburgh School of Medicine, PA, United States; National Institute on Drug Abuse, Intramural Research Program, Baltimore, MD, United States

**Keywords:** bereavement, acute grief, prolonged grief disorder, integrated grief, neuroimaging, functional MRI, task fMRI, resting-state functional connectivity, older adults

## Abstract

Bereavement is a near-universal experience in late life, yet only some older adults develop prolonged grief disorder (PGD). While most transition from acute grief (AG) to integrated (adaptive) grief, the neurobiological substrates underlying divergent trajectories are unclear. Emotion regulation dysfunction is hypothesized to play a central role in PGD pathogenesis, but longitudinal neuroimaging data in bereaved adults are lacking. This study aims to identify functional brain circuit measures of emotional regulation that predict pathological versus adaptive grief trajectories, aligning with the Research Domain Criteria (RDoc) framework for the Negative Valence System (Loss) construct. This single-site, 1-year longitudinal study aims to enroll 170 adults aged 50–89 years: 115 with AG and 55 age- and gender-equated non-bereaved participants. Participants will complete comprehensive psychiatric, neuropsychological, and psychosocial assessments, alongside neuroimaging both at study baseline and after 12 months. Functional neuroimaging includes resting-state fMRI, a face–shape matching task probing emotion processing, and a stop-signal task probing inhibitory control. Functional neuroimaging data are acquired using a harmonized Human Connectome Project protocol on a GE Signa Premier 3T MRI scanner. We present a comprehensive overview of the eligibility criteria, clinical study procedures, and neuroimaging protocol. Baseline findings from 103 AG and 40 non-bereaved participants thus far enrolled show that the groups are demographically matched and provide high-quality neuroimaging data and robust task performance. This study is among the first longitudinal neuroimaging investigations of AG in older adults and may identify early biomarkers of PGD risk, potentially guiding precision prevention and intervention strategies for bereaved older adults.

## 1 Introduction

Bereavement is a near-universal and often profoundly traumatic human experience. In 2024, over 62 million people died worldwide, including more than 3 million in the United States, leaving behind an average of 5 to 9 bereaved individuals per death of a spouse, parents, siblings, children, and other close relatives (Antonucci et al., 2004; Verdery et al., 2020). Older adults experience bereavement at higher rates than younger individuals, with nearly one-third of women and over 10% of men over 65 widowed (Gurrentz & Mayol-Garcia, 2021; Lobb et al., 2010). While most adapt and achieve integrated (adaptive) grief, a state of restored purpose and meaning in life without the deceased, approximately 10% of bereaved individuals develop prolonged grief disorder (PGD). PGD, a psychiatric condition recognized in both the DSM-5-TR and ICD-11, is distinct from other bereavement-related disorders such as late-life major depressive disorder (LLD). It is diagnosed at least 12 months post-loss and is characterized by intense yearning for and/or preoccupation with thoughts or memories of the deceased, emotion dysregulation (i.e., loneliness, emotional pain, anhedonia), and avoidance of loss-related reminders, resulting in marked functional impairment (Prigerson et al., 2021, 2022). The impact of PGD is profound, encompassing declines in physical health, cognitive function, quality of life, premature mortality, and suicide (Perez et al., 2018; Prigerson, Frank, et al., 1995; Prigerson et al., 1996, 1997; Stroebe et al., 2007; Szanto et al., 1997).

Despite its clinical significance, current diagnostic approaches cannot distinguish older adults who will transition from acute grief (AG) to integrated grief from those prone to prolonged grief trajectories. This critical gap limits early identification and the development of targeted treatments. Identifying neurobiological mechanisms underlying divergent grief trajectories is, therefore, critical to enable early detection, optimize existing interventions (Shear et al., 2014, 2016), and inform novel preventive strategies for at-risk individuals—bridging intervention science and clinical care.

### 1.1 Focus on emotion regulation: Rationale

Although not all encompassing, emotion regulation (ER) theory provides a compelling framework for understanding the neurobiological underpinnings of PGD pathogenesis. PGD is conceptualized as a stress response syndrome that arises when bereaved individuals fail to integrate the loss of an attachment figure into their mental representations, often due to maladaptive ER strategies such as avoidance (Shear & Shair, 2005; Shear et al., 2007). The death of a loved one is hypothesized to disrupt emotional processing and regulatory neuronal systems, among a range of cognitive and emotional processes (Eisma & Stroebe, 2021; Maccallum & Bryant, 2013). Accordingly, an efficient ER system likely facilitates grief adaptation by managing separation-related stress, modulating distress, and supporting eventual acceptance of loss. This longitudinal study follows older adults with AG to identify brain functional measures of emotion dysregulation associated with PGD versus integrated grief trajectories. Given the frequent overlap with depressive symptoms, we also aim to disambiguate shared and unique neural correlates of ER dysfunction underlying PGD and depression. We operationalize ER as a neurocognitive construct encompassing both emotional reactivity and regulatory control systems, rather than explicit emotion regulatory strategies per se.

The amygdala, a central hub of the emotion processing neural circuitry, detects and interprets emotionally salient stimuli. Together with its associated circuitry, it is involved in processing social cues and interpreting social interactions to assess potential threats or social rewards. It also influences decision-making processes by providing emotional input and plays a role in consolidating emotional memories. As such, the amygdala is of particular interest in grief research where it is implicated in clinical symptoms including sadness, separation distress, emotional memory storage, and threat detection (Goldin et al., 2005; Lemche et al., 2006; Phelps, 2006; Wang et al., 2005). Functional MRI (fMRI) studies show heightened amygdala activity in response to negative emotional stimuli in young bereaved individuals, both during acute (Freed et al., 2009) and prolonged grief (Fernandez-Alcantara et al., 2020). Notably, higher amygdala activity appears to differentiate PGD from major depression (Bryant et al., 2021) and integrated grief (Hwang et al., 2024).

To fully understand ER in general and specifically herein for late-life AG, one must also examine the role of prefrontal cortical regions that exert top–down control over the amygdala. These regions have been shown to modulate attentional and emotional responses in younger acutely grieving adults and may help distinguish intrusive from avoidant grief symptomatology (Freed et al., 2009), supporting the prefrontal control deficit theory of depression. However, fMRI studies of ER in older bereaved adults remain limited (Arizmendi et al., 2016; Freed et al., 2009; O’Connor, 2012). In LLD, which frequently co-occurs with grief, reduced prefrontal activity and diminished amygdala-prefrontal functional connectivity are documented (Naismith et al., 2012; Tadayonnejad & Ajilore, 2014). For instance, diminished frontal responses to emotional probes (Aizenstein et al., 2011; Brassen et al., 2008) and reduced amygdala resting-state functional connectivity (rsFC) with cognitive control regions (Li et al., 2015) support the prefrontal control deficit theory of depression. In contrast, our preliminary work suggests that higher amygdala–frontal rsFC is associated with worsening/persistent prolonged grief symptoms over time in older adults with AG (Chen et al., 2020).

Collectively, these findings lead us to hypothesize that distinct ER-related neural dysregulation may, at least in part, underlie PGD, underscoring the need to delineate neurobiological substrates of PGD pathogenesis in late-life AG. The task-based fMRI paradigms in this study assess neural responses to negative emotional processing and cognitive control, reflecting key neurocognitive components of regulatory capacity in acutely grieving older adults. By integrating neural, behavioral, and self-report measures in later-life AG, this project examines selected dimensions of the RDoC negative valence system (loss) construct: amygdala activation during the face–shape matching task indexes acute negative affect and threat responsivity; resting-state amygdala connectivity serves as a neural indicator of sustained separation distress; and stop-signal task performance reflects prefrontal-mediated inhibitory control relevant to top–down regulation of negative affect. The outcomes may identify novel brain functional measures of ER-relevant symptom dimensions that transcend diagnostic categories (PGD, depressive disorders, PTSD) and characterize long-term sequelae of AG.

### 1.2 Study objectives

Based on the extant literature and our preliminary data, our central hypothesis is that, in older adults with AG, greater ER circuit activity and amygdala–frontal functional connectivity, relative to non-bereaved comparison participants, reflect emotion dysregulation and represent early, critical imaging phenotypes associated with heterogeneous grief trajectories and the persistence of prolonged grief symptoms. We propose the following aims:

Aim 1. Characterize ER-related brain function in older adults with AG, compared with age- and gender-equated non-bereaved (NB) comparison participants, using comprehensive clinical assessments and functional neuroimaging (both task-based and resting-state).

Aim 2. Determine whether baseline ER-related brain functional measures can characterize longitudinal prolonged grief trajectories in AG.

Aim 3 (Exploratory). Assess whether sustained alterations in ER neurobiological substrates are associated with worsening or persistent prolonged grief symptom trajectories.

The primary objective of this paper is to outline the study protocol, including recruitment, screening procedures, inclusion/exclusion criteria, and detailed clinical and neuroimaging acquisition methods. We also describe the imaging preprocessing pipelines, quality control procedures, data management strategies, and data sharing plan. Additionally, we provide an overview of the preliminary functional imaging data and clinical characteristics of the enrolled sample at study baseline.

## 2 Methods

**Fig. 1.**
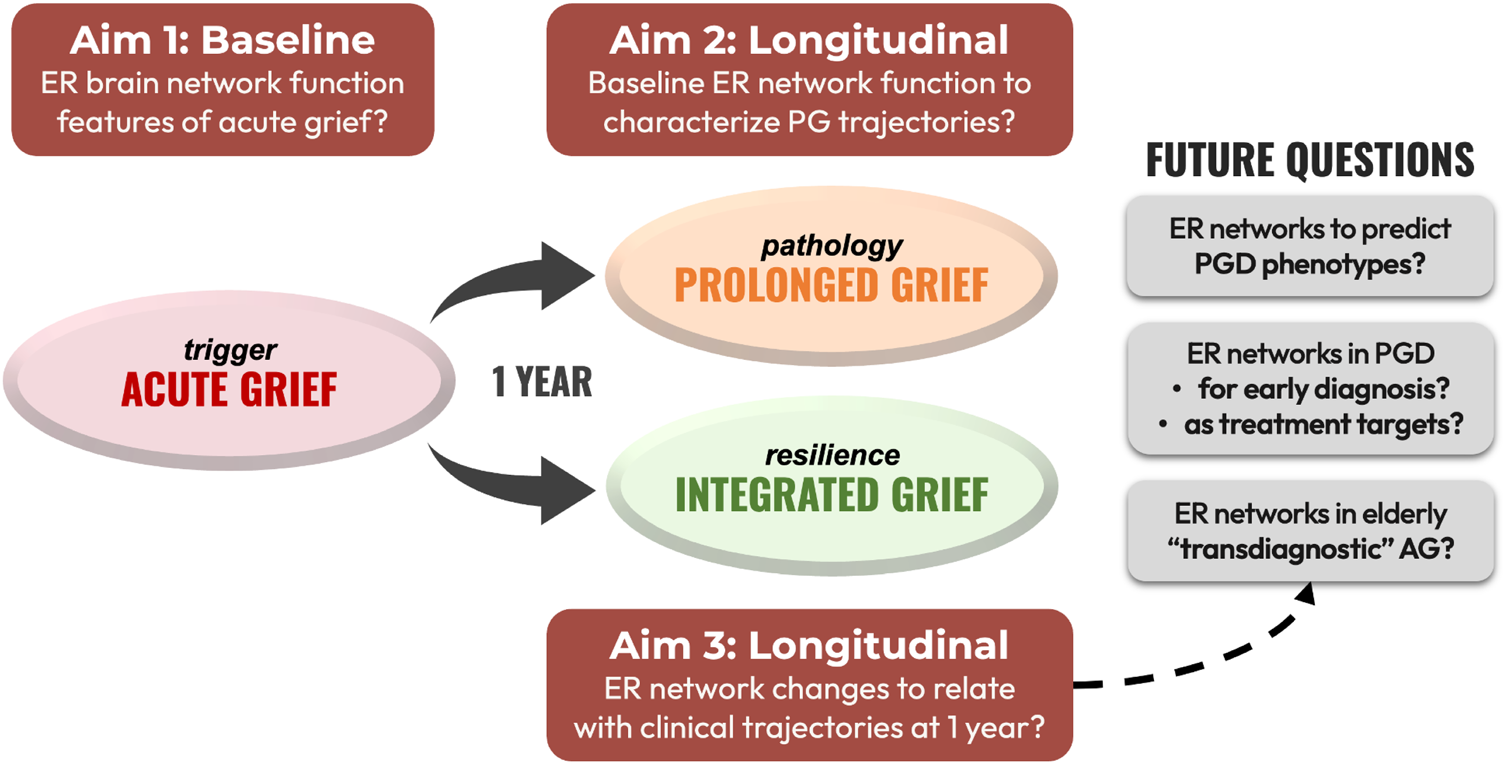
Research Framework. This study examines emotional dysregulation in the pathogenesis of prolonged grief disorder (PGD) by following acutely grieving (AG) older adults for 1 year. Abbreviation: ER, emotion regulation; PGD, prolonged grief disorder, AG: acute grief

This single-site longitudinal study was funded by the National Institute of Mental Health (R01MH122490) in February 2020. All study procedures were approved by the institutional review board of the Medical College of Wisconsin (MCW; protocol #36603) and conducted in accordance with ethical guidelines for human research. The overall framework and future directions are illustrated in Figure 1.

### 2.1 Study participants

#### 2.1.1 Enrollment goals and recruitment strategies

The goal of this study was to enroll a total of 170 participants, between ages 50 and 89 years, into 2 age- and gender-equated groups: (1) acute grief (AG; *n* = 115), and (2) currently non-bereaved comparison (NB; *n* = 55) groups. This enrollment was based on an anticipated 15% attrition, with a targeted final total sample of 145 participants (AG: *n* = 100, NB: *n* = 45) expected to complete the 12-month study.

Participants are recruited from the greater Milwaukee community through advertisements, brochures, flyers, media announcements, press releases, and presentations, including but not limited to community grief groups and hospice programs. Flyers are also placed in waiting areas of various clinics (e.g., primary care, geriatric medicine, psychiatry, palliative care). Enrolled participants receive prorated reimbursement following each study visit. Our multidisciplinary research team comprises clinicians, research coordinators, biostatisticians, signal processors, and imaging physicists (see Supplementary Fig. S1 for details).

Due to the coronavirus disease 2019 (COVID-19) pandemic, study procedures were temporarily suspended in March 2020. Upon resumption, enrollment proceeded cautiously given the older age of our target population. The study is ongoing; as of August 2025, we have enrolled 103 AG and 40 NB participants.

#### 2.1.2 Inclusion and exclusion criteria

Common inclusion criteria for all participants include age 50–89 years, adequate visual and auditory acuity, fluent in English, absence of dementia of any etiology, a Montreal Cognitive Assessment (MoCA) score ≥ 20 (Nasreddine et al., 2005, 2012), intact basic and instrumental activities of daily living, and a modified Hachinski Ischemic Scale ≤ 4. We focused on adults aged 50 years and older because bereavement is more commonly experienced later in life. Data suggest differences in task fMRI measures in older individuals, relative to their younger counterparts (Dhingra et al., 2020; Gunning-Dixon et al., 2003; St Jacques et al., 2009; Tessitore et al., 2005), including in those with attachment bereavement (McConnell et al., 2018; O’Connor et al., 2008). Potential pre-loss characteristics (e.g., lifetime trauma, psychiatric history, prior losses, higher medical illness burden), age-related characteristics (e.g., lower cognition, cerebral atrophy, cerebrovascular ischemia), and post-loss/associated contexts (e.g., circumstances of death, lower social support) are also unique among older adults and may differentially influence neural correlates of interest. We aim to capture age-dependent variability in the ER neural substrates. No minimum grief severity threshold was required for enrollment in the AG group, enabling capture of the full spectrum of acute grief responses as well as resilient and prolonged grief trajectories.

Common exclusion criteria include delirium/unstable medical conditions (i.e., Cumulative Illness Rating Scale-Geriatrics [CIRS-G] (Miller et al., 1992) score of 4 in any organ-specific category), lifetime history of neurological illnesses (seizures, stroke, dementia of any etiology, severe head injury, brain tumor, or neurosurgery), gross structural abnormalities on T1-weighted images at study baseline MRI scan as reported by a clinical neuroradiologist, lifetime history of bipolar or psychotic disorders, current alcohol/drug abuse or dependence, medications that can cause depression (reserpine, steroids, etc.), MRI contraindications, and acute suicidality (assessed using Columbia Suicide Severity Rating Scale [C-SSRS]) (Posner et al., 2011) or judged to be at serious suicide risk by the study physician.

To be included in the AG group, participants must have experienced a death of a loved one within the past 12 months. The broad enrollment window is designed to capture the full trajectory of AG from its earliest stages through the point of potential PGD diagnosis. Structured Clinical Interview for DSM-5 (SCID-5-RV) (2013) modules on mood, anxiety, and trauma- and stressor-related disorders are administered at baseline and end point. Those who meet DSM-5 criteria for these conditions are not excluded because these disorders often co-occur with AG and PGD. AG participants may be taking certain psychotropic medications, including antidepressant medications or low-dose benzodiazepines (i.e., lorazepam equivalent dose ≤ 2 mg/day), if doses are stable for at least 4 weeks prior to each MRI visit. This design reflects the longitudinal, observational nature of the study and aims to represent the acutely grieving cohort in the general clinical population, including its common comorbidities.

To be included in the NB group, participants must have no history of bereavement within the past 24 months and no lifetime diagnosis of any psychiatric illness. Those who had experienced death of a loved one > 2 years ago must score < 5 on the Brief Grief Questionnaire (BGQ) (Ito et al., 2012) for inclusion.

#### 2.1.3 Screening

Eligibility screening is conducted in two stages. The majority of inclusion and exclusion criteria are assessed during an initial telephone survey prior to scheduling the in-person baseline clinical visit, thereby minimizing participant burden. In rare cases, additional exclusionary information may emerge during the in-person assessment.

#### 2.1.4 Informed consent

At the study baseline clinical visit, written informed consent is obtained for all proposed study procedures. All enrolled participants require decisional capacity to provide informed consent, assessed using the UCSD Brief Assessment of Capacity to Consent (UBACC) (Jeste et al., 2007).

### 2.2 Study assessments

**Fig. 2.**
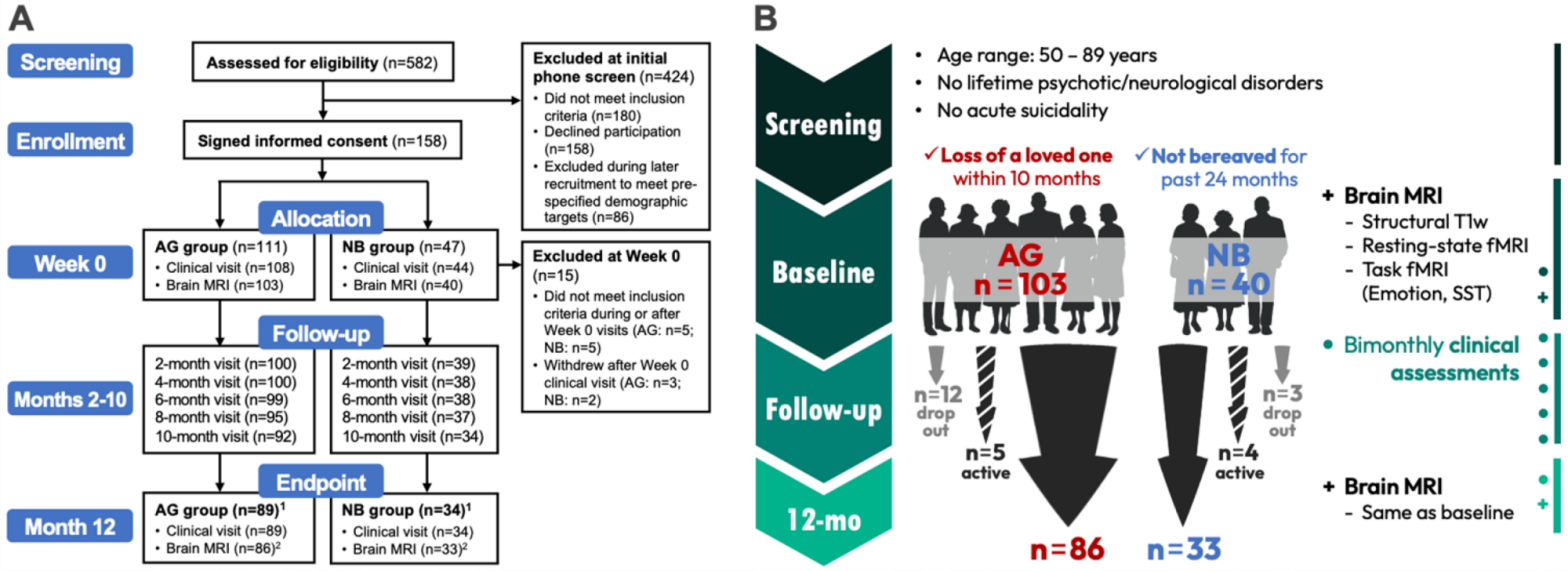
STROBE diagram and study design. (A) As of August 2025, 582 older adults were assessed for eligibility. Of these, 103 participants were enrolled in the acute grief (AG) group and 40 in the non-bereaved comparison (NB) group. Fifteen participants discontinued the study early, 9 remain active, and 119 have completed the 12-month MRI visit. (B) Brain MRIs are completed at study baseline (Day 2) and study end point (12-month post-study enrollment); clinical assessments are completed at study baseline (Day 1) and every 2 months thereafter. Abbreviation: AG, acute grief; NB, non-bereaved. ^1^Currently active in the study (*n *= 9; AG: *n *= 5; NB: *n *= 4). ^2^Attrition after Week 0 (clinical and brain MRI) visits (*n *= 5): lost to follow-up (AG: *n *= 6); dropped out (AG: *n *= 2; NB: *n *= 1); met exclusion criteria (AG: *n *= 4; NB: *n *= 2).

Each participant completes a 2-day baseline visit, with Day 1 for clinical assessments and Day 2 for brain imaging. Participants attend clinical assessment visits every 2 months thereafter. At the 12-month visit, participants undergo a second brain imaging session. A STROBE flow diagram and the overall study design are provided in Figure 2.

#### 2.2.1 Study baseline: Clinical (day 1)

This study employs well-validated multidimensional clinical assessment scales, with grief-specific scales consistent with those used in NIH-funded Complicated Grief studies (e.g., Optimizing Treatment of Complicated Grief; 2R01MH060783-06A1) (Table 1).

**Table 1.** Clinical assessment timetable.

Clinical Visit	Screening	Baseline	2 mo	4 mo	6 mo	8 mo	10 mo	12 mo
Telephone survey	●							
Informed consent		●						
**Primary grief-related self-report measures**								
ICG^a^		●	●	●	●	●	●	●
PG-13/PG-13-R^a^		●	●	●	●	●	●	●
YSL^a^		●	●	●	●	●	●	●
GRAQ^a^		●	●	●	●	●	●	●
IES-R		●			●			●
UCLA-3		●	●	●	●	●	●	●
**Other psychiatric self-report measures**								
PHQ-9		●	●	●	●	●	●	●
PCL-5		●	●	●	●	●	●	●
MASQ		●	●	●	●	●	●	●
Insomnia Severity Index		●			●			●
**Clinician-administered assessments**								
SCID-5-RV^b^		●			○			○
SCI-CG^a,c^					○	○	○	●
HDRS-17		●			●			●
HAM-A		●			●			●
C-SSRS		●	●	●	●	●	●	●
ATHF		●	●	●	●	●	●	●
**Medical, cognition, functional measures**								
CIRS-G		●			●			●
Neuropsychological battery^d^		●						●
**Other self-report measures**								
SAAM		●			●			●
Duke Social Support Index		●		●		●		●
Lubben Social Network Scale		●		●		●		●
CTQ			●					
Big 5 Inventory			●					

Closed circles represent assessments completed by all participants (with the exception of grief specific measures marked with superscript a, which were completed by AG participants only).

a Only in AG.

b As needed (indicated by open circles).

c PG-13-R >=20 at the closest visit after crossing the 12-month anniversary as well as 12-month end point visit (indicated by open circles).

d Includes National Adult Reading Test, Montreal Cognitive Assessment, Dementia Rating Scale-2, Immediate and Delayed Paragraph Recall, Boston Naming Test, Category Fluency, Trails Making Test, Digit Symbol Substitution Test, Stroop Color Word Test, Clock Drawing Test, Physical Self-Maintenance Scale, and Lawton Instrumental Activities of Daily Living Scale.

ICG: Inventory of Complicated Grief, PG-13-R: Prolonged Grief 13 questions Revised, YSL: Yearning in Situations of Loss, GRAQ: Grief-Related Avoidance Questionnaire, IES-R: Impact of Event Scale–Revised, UCLA-3: 20-item University of California Los Angeles Loneliness scale-version 3, PHQ-9: Patient Health Questionnaire, PCL-5: Post-traumatic stress disorder Checklist for DSM-5, MASQ: Mood and Anxiety Symptom Questionnaire, SCID-5-RV: Structured Clinical Interview for DSM-5, SCI-CG: Structured Clinical Interview for Complicated Grief, HDRS-17: 17-item Hamilton Depression Rating Scale, HAM-A: 14-item Hamilton Anxiety scale, C-SSRS: Columbia Suicide Severity Rating Scale, ATHF: Antidepressant Treatment History Form, CIRS-G: Cumulative Illness Rating Scale-Geriatrics version, SAAM: State Adult Attachment Measure, CTQ: Childhood Trauma Questionnaire.

##### 2.2.1.1 Semi-structured clinical interview

After consenting, a study clinician conducts a clinical interview, including the Structured Clinical Interview for DSM-5 (SCID-5-RV; overview and modules on screening, current and past mood, anxiety, trauma- and stressor-related, and substance use disorders, psychotic symptoms), 17-item Hamilton Depression Rating Scale (HDRS-17) (Hamilton, 1980), 14-item Hamilton Anxiety scale, HAM-A (Hamilton, 1959), the Columbia Suicide Severity Rating Scale (Posner et al., 2011), Cumulative Illness Rating Scale-Geriatrics version (CIRS-G) (Miller et al., 1992), the modified Hachinski Ischemic Scale, medication review and psychotropic medication treatment history (using antidepressant treatment history form [ATHF]) (Prudic et al., 1996; Sackeim, 2001), and a neurological examination.

Research coordinators assess MRI eligibility, collect prescription and over-the-counter medication information, and obtain vital signs. Additional information collected includes history of trauma and bereavement, family history of mood disorders, living alone status, and date, cause, and circumstances of the loved one’s death.

In addition, the research coordinators complete a neuropsychological battery, consistent with our prior study in AG (Hoffmann et al., 2024): Premorbid intelligence is measured using the National Adult Reading Test (Nelson, 1982); global cognitive function using the MoCA and the Dementia Rating Scale-2 (DRS-2) (Lucas et al., 1998); memory using the Immediate and Delayed Paragraph Recall (Wechsler, 1987); language using the Boston Naming Test (Kaplan et al., 1983) and Category Fluency (animals) (Butters et al., 1987); executive function using the Trails Making Test B (Reitan, 1955) and the Clock Drawing Test (Freedman et al., 1994); attention and information processing speed using the Digit Symbol Substitution Test (DSST) (Wechsler, 2008) and Trails Making Test A (Reitan, 1955); inhibitory control using the Stroop Color Word test (Golden, 1978). The Physical Self-Maintenance Scale for basic activities of daily living and Lawton Instrumental Activities of Daily living Scale are also completed (Lawton & Brody, 1969).

##### 2.2.1.2 Self-report questionnaires

Grief-specific symptoms are assessed in the AG participants: (1) *Overall grief symptoms*. The Inventory of Complicated Grief (ICG) is a 19-item questionnaire with excellent psychometric properties and reliably assess complicated grief (a term used to define prolonged grief before DSM-5-TR inclusion) symptoms (Prigerson, Maciejewski, et al., 1995). The ICG has been previously utilized in PGD clinical trials (Shear et al., 2016), with a score of ≥ 30 suggestive of elevated complicated grief symptoms. Following the inclusion of PGD in the DSM-5-TR, we also administer the Prolonged Grief-13-item-revised (PG-13-R) scale, which maps onto the DSM-5-TR PGD criteria. The PG-13-R demonstrates high internal consistency and reliability (Prigerson et al., 2021). Note that the PG-13-R scale was introduced in this study on April 16, 2021, after the study had commenced; prior to that, the older version, the PG-13 scale, was used (PG-13-R is used for notational convenience throughout this manuscript). (2) *Yearning*. The 21-item Yearning in Situations of Loss (YSL)-Bereaved scale (O’Connor & Sussman, 2014) has good discriminant and convergent validity to assess yearning. (3) *Intrusive thoughts*. The Impact of Event Scale—Revised (IES-R) (Baumert et al., 2004), which shows high internal consistency and test–retest reliability, is used to assess intrusive thoughts via the intrusion subscale. (4) *Avoidance of reminders of the loss*. Grief-Related Avoidance Questionnaire (GRAQ) (Shear et al., 2007) is a 15-item questionnaire to rate the degree of avoidance of specific situations related to bereavement. The *Loss Summary* (Shear et al., 2016), providing an overview of the participant’s loss history, is also completed.

In addition, both the AG and NB participants complete the 20-item *UCLA loneliness scale-version 3 (UCLA-3)* (Russell, 1996) to assess loneliness, the *Patient Health Questionnaire (PHQ-9)* (Kroenke et al., 2001) for depressive symptoms, *PTSD Checklist for DSM-5 (PCL-5)* (Blevins et al., 2015; Bovin et al., 2016) for PTSD symptoms, the *Insomnia Severity Index* (Bastien et al., 2001), the *Mood and Anxiety Symptom Questionnaire (MASQ)-short form* (Watson et al., 1995) for depressive mood, anxious arousal, and hedonic capacity, the *State Adult Attachment Measure (SAAM)* for attachment styles (Gillath et al., 2009), and the *Duke Social Support Index* (Koenig et al., 1993) and *Lubben Social Network Scale* (Lubben et al., 2006) for social support.

#### 2.2.2 Study baseline: Magnetic resonance imaging (day 2)

MRI data are acquired at the MCW Center for Imaging Research on a GE SIGNA Premier 3T scanner using a Nova 32-channel head coil. We have implemented and harmonized the HCP protocol on this scanner, offering improved signal-to-noise ratio (SNR) and enhanced parallel and multi-slice imaging capabilities compared with previous GE scanners. This project’s multiband fMRI acquisition follows established HCP protocols using GE 3-Tesla scanners: Mapping Connectomes for Disordered Emotional States (NIMH U01 MH109985) and Adolescent Brain Cognitive Development (ABCD) (NIDA U01 DA041025) (Casey et al., 2018; Tozzi et al., 2020).

During the visit, participants undergo the following MRI scans: (1) T1-weighted anatomical, (2) T2-weighted anatomical, (3) resting-state BOLD (two runs), (4) emotional face-matching fMRI task, (5) stop signal fMRI task (two runs). The two task-based fMRI paradigms were selected to probe bottom–up emotional reactivity and top–down general inhibitory control—neurocognitive components of ER capacity implicated in PGD—while maintaining feasibility and minimizing participant burden in an older adult sample. Before each fMRI run, spin-echo field maps are acquired. Prior to scanning, participants complete a practice run of each task on a computer outside the scanner. Detailed scan acquisition parameters are provided in Table 2.

**Table 2.** MRI scan parameters.

Scan type	T1-weighted	T2-weighted	Spin-echo fieldmap^a^	Multiband fMRI		
Sequence	MPRAGE	Sag CubeT2	-	-		
TR (ms)	2500	2500	7400	800		
TE (ms)	3	90.4	80	30		
TI (ms)	900	-	-	-		
Flip angle (degrees)	8	90	90	50		
Voxel size (mm^3^)	1.0 x 1.0 x 1.0	0.5 x 0.5 x 0.8	1.69 x 1.69 x 2.4	2.4 x 2.4 x 2.4		
Field of view (mm^2^)	256 x 230.4	256 x 230.4	216 x 216	216 x 216		
% Phase field of view	90%	90%	100%	100%		
Matrix size	256 x 256	512 x 512	128 x 128	90 x 90		
Number of slices	184	230	60	60		
Pixel bandwidth (Hz/pixel)	244.141	244.141	3906.25	5555.56		
Parallel imaging	x2	x2	-	-		
Multiband acceleration	-	-	-	6		
Task	-	-	-	Resting-state	Emotion task	SST task
Number of volumes	-	-	2	563	525	450
Acquisition time	5:19	4:16	0:15	15:00^b^	7:00	12:00^b^

a Two runs with the opposite phase-encoding directions were acquired prior to each fMRI scan.

b Acquired over two runs of equal length.

##### 2.2.2.1 Resting-state fMRI (Rs-fMRI)

Participants are instructed to close their eyes without falling asleep. Two 7.5-minute runs are acquired with opposite phase encoding directions.

##### 2.2.2.2 Emotional face–shape matching paradigm

This block design task (Hariri et al., 2002) robustly activates the amygdala, dorsolateral prefrontal cortex (dlPFC), insula, and the dorsal anterior cingulate cortex (ACC), and has been utilized in a prior PGD study (Hwang et al., 2024). This paradigm was included to measure neural responses to generalizable negative emotional stimuli, reflecting affective components of the RDoC Loss construct.

The task comprises blocks of trials containing either emotional expressive faces (fearful, angry, surprised, or neutral expression) or oval shapes. There are a total of nine blocks, each block containing six trials, with alternating blocks of faces and shapes. Each face or shape trial is presented for 4 seconds. In each trial, participants are asked to indicate which of the two stimuli (faces or shapes) presented at the bottom of the screen match the probe stimulus at the top. Accuracy (% correct responses) and reaction time are collected. The primary contrast of interest is the fearful plus angry faces (i.e., the two negative affect faces) minus shapes, with fear minus shapes and angry minus shapes as secondary contrasts of interest.

##### 2.2.2.3 Stop signal task

The Stop Signal Task (SST) probes the cognitive construct of response inhibition, a core executive control function required to suppress inappropriate or impulsive actions, including emotional reactions that may interfere with goal-directed behavior (Verbruggen & Logan, 2008). In the current context, the SST serves as a behavioral index of cognitive control that may facilitate regulation of negative affect in the context of loss, linking to the regulatory aspects of the RDoC loss domain. It engages core brain regions of impulsivity and impulse control, including the inferior frontal gyrus (IFG), pre-supplementary motor area (pre-SMA), anterior insula, dorsal ACC, dlPFC, and basal ganglia.

The SST used herein is identical to the one used in the ABCD study (Casey et al., 2018). Each run consists of 180 trials, where participants respond using a 2-button response panel corresponding to the direction of the arrow on the screen. Participants are instructed to respond as quickly and accurately as possible. The “Go” stimulus remains on the screen for 1000 ms or until a response is made, whichever comes first. One-sixth of the trials are Stop trials. In these trials, the Go stimulus is followed unpredictably by a Stop signal, presented as an upward-pointing arrow for 300 ms. The Go stimulus remains visible for the duration of the Stop Signal Delay (SSD) or until a response is made, whichever occurs first. If the SSD exceeds 700 ms, the Stop Signal (SS) duration is reduced such that the combined SSD and Stop Signal duration equals 1,000 ms. The SSD is dynamically adjusted using a staircase tracking algorithm to maintain an inhibition success rate of approximately 50%, increasing after successful stops and decreasing after unsuccessful stops.

A performance criteria to calculate the Stop task performance and to ensure adequate task engagement have been proposed for the ABCD data (Garavan et al., 2022). Given our older sample, we applied two sets of exclusion criteria: one tailored to older populations and a second set based on Garavan et al.’s adolescent cohort. Here, we report data using the modified ABCD performance exclusion criteria (*n* = 126) and include results using the ABCD performance criteria in the Supplementary Material (*n* = 112; see Supplementary Methods 1 for more details).

### 2.3 Longitudinal study procedures

#### 2.3.1 Clinical data

To decrease participant burden during the study baseline visit, the *Childhood Trauma Questionnaire (CTQ)* (Bernstein et al., 1997, 2003) and the *Big Five Inventory* (John & Srivastava, 1999) are completed at the 2-month follow-up visit.

##### 2.3.1.1 Follow-up (every 2 months) visits

The following self-report scales are completed during each clinical visit by all participants: PHQ-9, PCL-5, UCLA-3, and MASQ. AG participants also complete the ICG, PG-13-R, YSL, and GRAQ scales. Additionally, suicidality is assessed using the C-SSRS, and psychotropic medication changes are tracked using the ATHF (if needed).

##### 2.3.1.2 Six-month and 12-month follow-up visits

The study clinician completes HDRS-17, HAM-A, CIRS-G, and SCID-5 if needed. The participants also complete the IES-R, Insomnia Severity Index, and SAAM during these visits.

##### 2.3.1.3 Other longitudinal assessments

At the first follow-up visit occurring after the 12-month anniversary of the loss, Structured Clinical Interview for Complicated Grief (SCI-CG) is administered to all participants who score 20 or higher on the PG-13-R. The SCI-CG and neuropsychological battery are repeated at the 12-month end point visit. Additionally, the social support measures (Duke Social Support Index, Lubben Social Network Scale) are repeated at 4-, 8-, and 12-month follow-up visits.

#### 2.3.2 MRI data

At the 12-month visit, participants complete the same MRI scan protocol as at study baseline. The overall study design is summarized in Figure 2.

### 2.4 Imaging data pre-processing

Images are preprocessed using *fMRIPrep* (v22.0.2) (Esteban et al., 2019). As part of the *fMRIPrep* workflow, structural T1w and T2w images are preprocessed with *FreeSurfer* (v7.3.2) (Fischl et al., 2002). Skull-stripped brain images are then spatially normalized to the standard MNI152 nonlinear asymmetric template (Fonov et al., 2011). All preprocessing scripts are executed within a *Singularity* container (v3.7.1) (Kurtzer et al., 2017) with the *ICA-AROMA* (Independent Component Analysis - Automatic Removal of Motion Artifacts) (Pruim et al., 2015) option enabled. The detailed *fMRIPrep* boilerplate is provided in Supplementary Methods 2.

After structural MRI preprocessing with *FreeSurfer*’s *recon-all* workflow, the multiband fMR images are corrected for slice time differences and susceptibility distortion using paired field maps and then aligned to the T1w reference image. The first 10 time points are discarded to account for T1-saturation effects. Subsequently, *ICA-AROMA* is used to identify nuisance signals likely attributed to motion artifacts. The nuisance signals, together with six anatomical CompCor (Component-based noise Correction) (Behzadi et al., 2007) time series and low-frequency cosine signals (high-pass filter; 0.0078 Hz or 1/128 s), are regressed out from the fMR images using the *NiLearn* library (v0.10.1) in *Python* (v3.9.1).

For both resting-state and emotion task images, a 0.1 Hz low-pass filter and spatial smoothing (full width at half maximum = 6.0 mm) are additionally applied. These corrections are not applied to the SST images, given their event-related design.

### 2.5 Retention strategies

AG participants have access to the grief clinic and the geriatric psychiatry clinics staffed by MCW psychiatry providers. They also have access to the PI and the on-call psychiatrist paging system throughout the entire study duration. Supplementary Methods 3 detail the suicide risk monitoring procedure.

Study staff contact participants 1 day before each visit to provide appointment reminders and update contact information at each follow-up. Upon request, participants receive a summary of the baseline and study end point visits.

### 2.6 Data management, accessibility, and repository

Clinical data are managed using Research Electronic Data Capture (REDCap), a secure web-based application supporting validated data entry, audit trails, and data import/export. Imaging data are archived, shared, and processed using XNAT (Extensible Neuroimaging Archive Toolkit). Only personnel with study approval can access these data. Neuroimaging and clinical data will be made available through the NIMH Data Archive (NDA) in accordance with the National Institute of Mental Health data sharing policy. No HIPAA (Health Insurance Portability and Accountability Act) protected information is transferred to the NDA.

### 2.7 MRI quality assurance

Detailed MRI quality assurance procedures are provided in Supplementary Methods 4. Briefly, daily phantom-based quality assessments following FBIRN (Functional Biomedical Informatics Research Network) standards monitor scanner performance and signal stability. In the case of MRI software updates, a staged validation protocol ensures that scan parameters and image quality remain unchanged. Post-acquisition quality checks include automated and visual assessments of SNR, head motion, field of view, and signal loss. Temporal SNR (tSNR) is calculated on *fMRIPrep* outputs prior to ICA-AROMA denoising and its consistency is compared with prior validated MB6 datasets (Hagler et al., 2019).

Overall head motion during the scan is quantified using mean frame-wise displacement (FD). For both task and resting fMRI data, participants with a mean FD > 0.5 mm are considered as excessive head motion outliers and are removed from further analyses (Kim et al., 2023; Power et al., 2012). For rs-fMRI, those participants with 30% or more time points with FD > 0.5 mm are additionally excluded in the group analyses to ensure at least 10 minutes of scan data for reliable rsFC estimation (Birn et al., 2013).

### 2.8 Statistical approach and analytic plans

Based on the established role of the amygdala, dorsal ACC, anterior insula, and dlPFC in ER, these regions will serve as *a priori* ROIs in both task-based and resting-state analyses, including seed-based functional connectivity analyses. This targeted approach is theoretically grounded and limits the multiple comparisons burden for primary hypothesis testing. Exploratory whole-brain analyses will also be conducted to allow detection of neural effects beyond these *a priori* regions, with appropriate corrections for multiple comparisons applied.

A key analytic priority across all three aims is isolating grief-specific neural variance from the effects of comorbid depression, anxiety, and PTSD, whose neural substrates may overlap with those hypothesized for PGD. To address this, participants’ comorbid symptom severity scores will be entered as covariates in all primary neuroimaging analyses. Prior to inclusion, all covariates and independent variables will be screened for multicollinearity to ensure model stability. While we cannot definitively dissociate PGD from LLD without a non-bereaved depressed control group, the use of symptom-specific covariates allows identification of neural variance specifically associated with grief severity.

For Aim 1, comparisons between AG and NB groups will be conducted using analysis of covariance (ANCOVA) to examine early brain circuit changes during the acute phase of bereavement. For Aim 2, grief symptom severity scores (PG-13-R) will serve as the primary continuous outcome in the linear mixed-effects (LME) models, given the expected low frequency of PGD diagnoses. For Aim 3, longitudinal changes in neuroimaging measures and grief symptoms will also be examined using LME models, which accommodate repeated measures, individual variability in baseline symptom severity, and differences in time since loss at enrollment.

Age, gender, and time since loss will be included as covariates in all primary analyses. Where sample size permits, exploratory subgroup analyses will compare AG participants with and without comorbid depression to examine whether hypothesized ER-related neural differences are present across grief subgroups or are primarily driven by co-occurring depression. Additionally, age, time since loss, and baseline depressive symptoms will be explored as moderators of brain–clinical associations. These analyses are hypothesis generating and will be interpreted accordingly.

## 3 Results

### 3.1 Clinical characteristics

Table 3 summarizes the demographic and clinical characteristics of the sample, as well as statistics comparing the AG and NB groups who were enrolled as of August 2025 and completed both study baseline visits. As expected, the AG group exhibited significantly higher depression, anxiety, PTSD symptoms, and loneliness than the NB comparison group (all *p* < 0.001).

**Table 3.** Study participant demographics and clinical characteristics.

Group	Acute Grief (*n* = 103)	Non-bereaved Comparison (*n* = 40)	*p* ^a^
Age [years]	64.5 ± 8.9 (50 - 83)	66.2 ± 7.4 (51 - 87)	0.26
Sex [female/male]	76 / 27	26 / 14	0.40
Education [years]	16.1 ± 2.6 (12 - 30)	16.8 ± 2.6 (13 - 25)	0.15
Race [White/Black/other]	90 / 4 / 9	37 / 1 / 2	0.68
Relationship with deceased [spouse/child/parent/other]	42 / 9 / 29 / 23	-	-
Type of loss (illness > 1 month/illness < 1 month/violent^b^)	62 / 33 / 8	-	-
Time since loss [days]	136.3 ± 85.1 (16 - 323)	-	-
PG-13-R^c^	24.3 ± 7.8 (11 - 44)	-	-
YSL	54.4 ± 21.9 (24 - 102)	-	-
IES-R intrusion	1.3 ± 1.0 (0 - 4)	-	-
GRAQ	12.6 ± 11.7 (0 - 56)	-	-
ICG anger	2.7 ± 2.4 (0 - 8)	-	-
UCLA loneliness	42.1 ± 10.9 (20 - 74)	31.6 ± 7.3 (20 - 45)	<0.001
HDRS-17	10.3 ± 5.8 (1 - 27)	1.8 ± 1.4 (0 - 5)	<0.001
HAM-A	6.8 ± 4.3 (0 - 23)	1.5 ± 1.4 (0 - 6)	<0.001
PCL-5	19.3 ± 15.0 (0 - 67)	2.6 ± 3.4 (0 - 13)	<0.001
MoCA	26.8 ± 2.2 (19 - 30)	27.0 ± 1.7 (22 - 30)	0.64
CIRS-G without psych	4.6 ± 3.4 (0 - 17)	3.5 ± 2.8 (0 - 12)	0.060
Current psychiatric disorder [depression/anxiety/trauma]	37 / 13 / 33	0 / 0 / 0	-
Past psychiatric disorder [depression/anxiety/trauma]	39 / 15 / 7	1 / 0 / 0	-
Current antidepressant treatment [SSRI+/other combination therapy/SSRI/other monotherapy/none]^d^	10 / 1 / 14 / 9 / 69	0 / 0 / 0 / 2 / 34	-

a *p*-values are from two-sample t-tests or chi-square tests, as appropriate.

b Violent causes of death include losses from accident, suicide, or homicide.

c The PG-13-R scale was incorporated mid-study when it became available. Baseline scores for the initial 16 participants were obtained using the original PG-13, and for the latter 87 participants, the PG-13-R was used.

d Thirteen of 103 AG participants (12.6%) were taking benzodiazepines at study baseline.

ICG: Inventory of Complicated Grief, PG-13-R: Prolonged Grief 13 questions Revised, YSL: Yearning in Situations of Loss, GRAQ: Grief-Related Avoidance Questionnaire, IES-R: Impact of Event Scale–Revised, UCLA Loneliness: University of California Los Angeles Loneliness scale-version 3, PCL-5: Post-traumatic stress disorder Checklist for DSM-5, HDRS-17: 17-item Hamilton Depression Rating Scale, HAM-A: 14-item Hamilton Anxiety scale, CIRS-G: Cumulative Illness Rating Scale-Geriatrics version, SSRI: Selective Serotonin Reuptake Inhibitor.

**Fig. 3.**
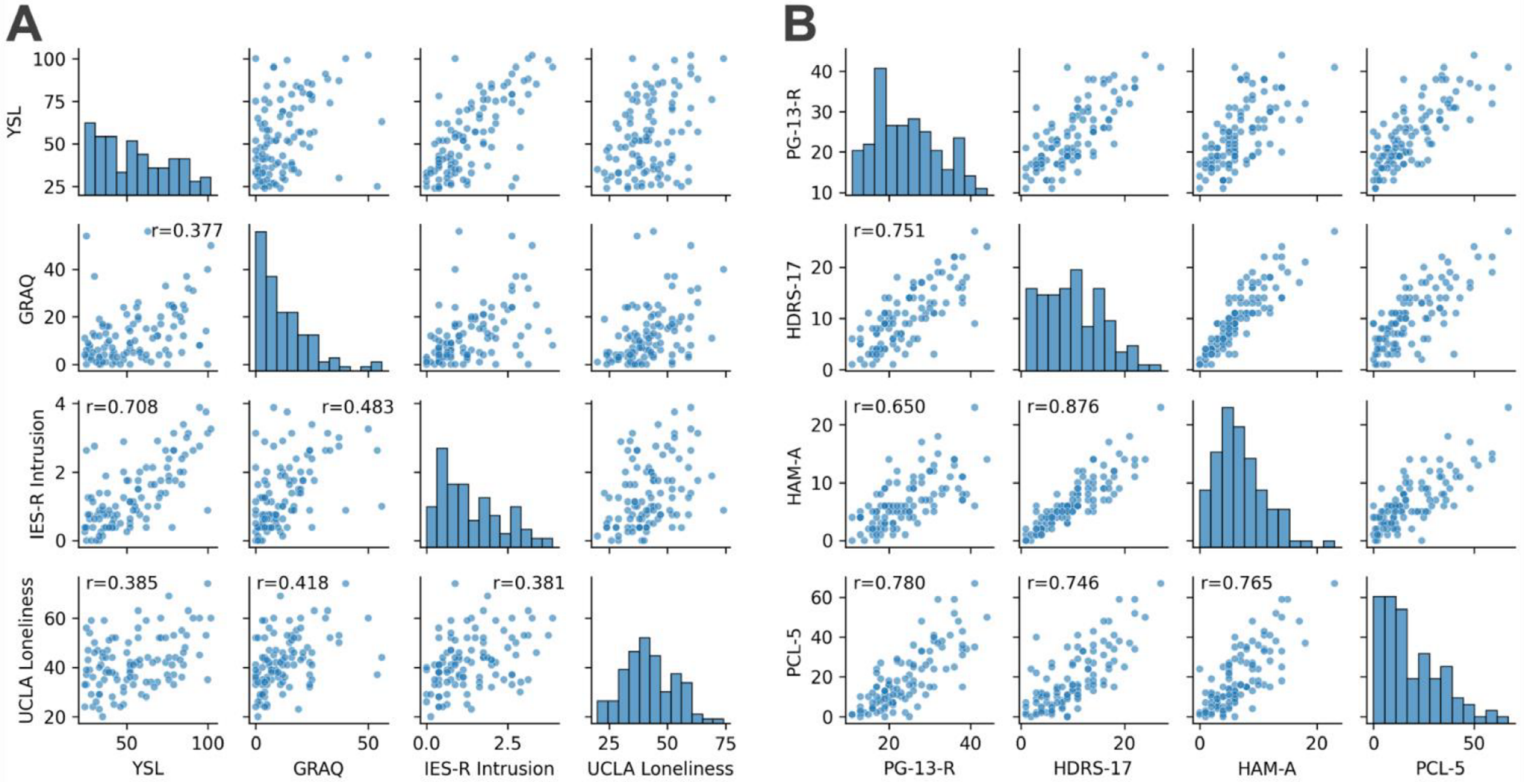
Relationships between clinical symptoms in acute grief. (A) Relationships between yearning (YSL), avoidance (GRAQ), intrusion (IES-R Intrusion), and loneliness (UCLA Loneliness) (*n *= 103). (B) Relationships between overall grief symptom (PG-13-R) and common clinical comorbidities (*n *= 103). Abbreviation: GRAQ, Grief-Related Avoidance Questionnaire; HAM-A, 14-item Hamilton Anxiety scale; HDRS-17, Hamilton Depression Rating Scale; IES-R, The Impact of Event Scale – Revised; PCL-5, Post-traumatic stress disorder Checklist for DSM-5; PG-13-R, Prolonged Grief-13-item-revised; UCLA Loneliness, 20-item University of California Los Angeles Loneliness scale-version 3; YSL, Yearning in Situations of Loss.

Figure 3A shows the relationship between the grief-specific symptoms of interest in the AG sample: yearning, intrusive thoughts, avoidance, and loneliness. The strongest correlation was found between yearning and intrusion (*r* = 0.708), while the weakest correlation was found between yearning and avoidance (*r* = 0.377). Figure 3B shows relationships between PG-13-R and comorbid psychiatric symptoms: Depressive, anxiety, and PTSD symptoms. PG-13-R shows strong correlations with PCL-5 (*r* = 0.780), HDRS-17 (*r* = 0.751), and HAM-A (*r* = 0.650) scores.

### 3.2 Baseline functional imaging

Neuroimaging data are reported for the entire cohort only. We have not conducted AG vs NB neural measure comparisons at this interim stage to avoid potential bias; these will be performed once recruitment is complete.

#### 3.2.1 Rs-fMRI

**Fig. 4.**
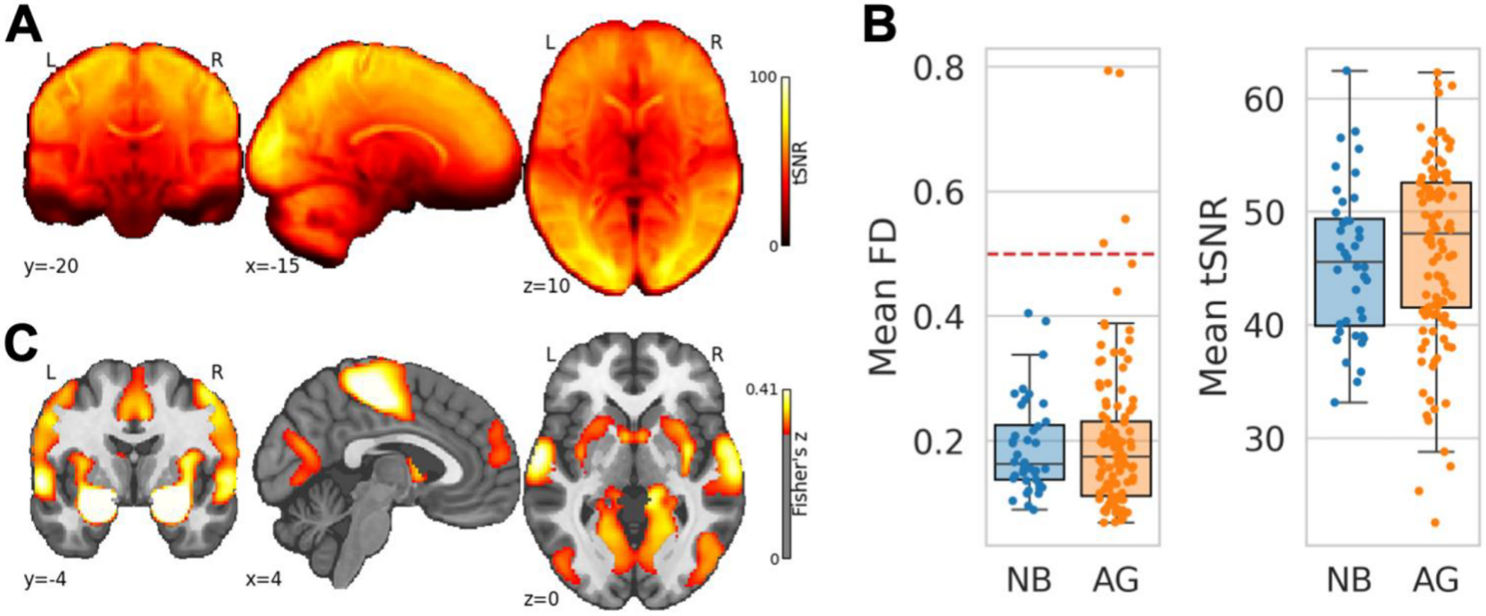
Summary of the resting-state fMRI. (A) Average temporal signal-to-noise ratio (tSNR) map before outliers were excluded (*n *= 143). (B) No significant group differences in mean tSNR (*p *= 0.65) and mean frame-wise displacement (FD) (*p *= 0.28) before motion outliers were excluded (*n *= 143). Red horizontal dotted line indicates a mean FD of 0.5 mm; participants above this threshold were labeled as motion outliers. (C) Amygdala connectivity after excluding motion outliers (*n *= 136). For visualization purposes only, the map displays Fisher’s z scores with a threshold of 0.3. Abbreviations: FD: frame-wise displacement; tSNR: temporal signal-to-noise-ratio; NB: non-bereaved comparison group; AG: acute grief

The Rs-fMRI data showed a mean tSNR of 46.03 ± 7.80 (Fig. 4A) and a mean FD of 0.20 ± 0.12 mm. There was no significant differences between groups for mean tSNR (AG: 46.20 ± 8.20; NB: 45.59 ± 6.66; *t*-test *p* = 0.65) and mean FD (AG: 0.21 ± 0.13 mm; NB: 0.19 ± 0.08 mm; *p* = 0.28) (Fig. 4B). Four AG participants exhibited a mean FD > 0.5 mm, while two additional AG and one NB participant had 30% or more time points with FD > 0.5 mm; all were excluded from further analyses. Using the bilateral amygdala masks from the Harvard–Oxford atlas (Jenkinson et al., 2012), rsFC maps were computed per individual for the remaining 136 participants. Across the full sample, the amygdala seed showed significant positive connectivity with bilateral hippocampi, parahippocampal gyri, putamen, temporal poles orbitofrontal cortex, medial prefrontal cortex, visual cortex, precentral and postcentral gyri, and left insula, consistent with prior reports of amygdala resting-state functional connectivity patterns from ours and other groups (Li et al., 2015; Roy et al., 2009; Xiao et al., 2018) (Fig. 4C).

#### 3.2.2 Face–shape matching task

**Fig. 5.**
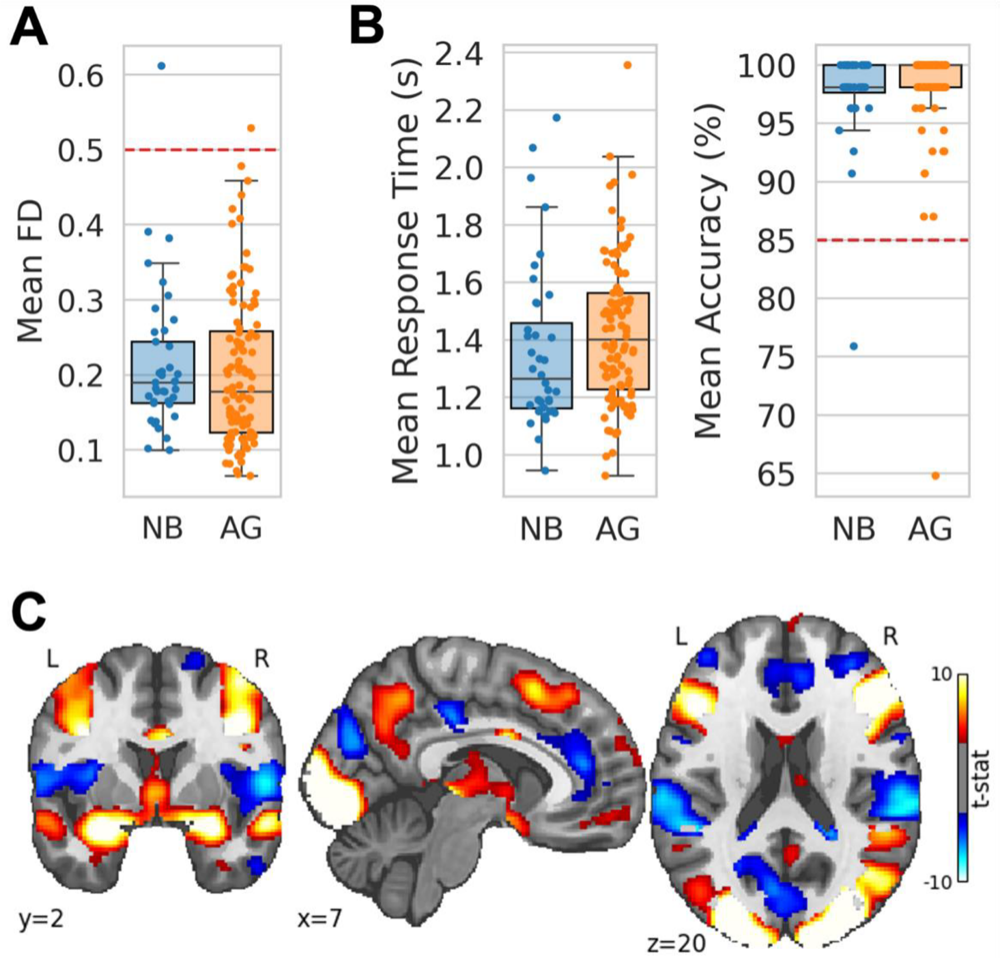
Summary of the emotion task fMRI. (A) No significant group differences in mean frame-wise displacement (FD) before outlier exclusion (*p *= 0.61; *n *= 141). Red horizontal dotted line indicates a mean FD of 0.5 mm; participants above this threshold were labeled as motion outliers. (B) Mean task response time and accuracy (*n *= 141). Red dotted line indicates a mean accuracy of 85%; participants below this threshold were labeled performance outliers. No participant was excluded based on the mean response time. (C) One-sample activation map of negative emotion contrast after excluding motion and performance outliers (fear plus anger minus shapes; *n *= 137). The statistics were cluster corrected with a voxel-wise *p* < 0.001 and cluster α = 0.05. Abbreviations: FD: frame-wise displacement; NB: non-bereaved comparison group; AG: acute grief.

One AG and one NB participants did not complete the emotion task fMRI. The emotion task data for the remaining 141 participants yielded a mean tSNR of 45.67 ± 8.13 (Supplementary Fig. S2A & S2B) and a mean FD of 0.21 ± 0.10 mm. There were no significant group differences between the mean tSNR (AG: 46.01 ± 8.43; NB: 44.81 ± 7.19; *p* = 0.41) or mean FD (AG: 0.21 ± 0.10 mm; NB: 0.22 ± 0.10 mm; *p* = 0.61) (Fig. 5A). One AG and one NB participants exhibited excessive head motion during the scan. Additionally, one AG and one NB participants performed below 85% task accuracy threshold; all were excluded from further analyses (Fig. 5B). Task accuracy and response time were statistically comparable between groups among the remaining 137 participants (*p* > 0.05). Negative emotion contrast maps (fear plus anger minus shapes) were generated for the remaining 137 participants. Significant task activation in the inferior frontal gyrus, amygdala, thalamus, supramarginal gyrus, and visual areas was observed across the entire cohort, consistent with prior reports using this paradigm (Hariri et al., 2002; Savage et al., 2024) (Fig. 5C).

#### 3.2.3 Stop signal task (SST)

**Fig. 6.**
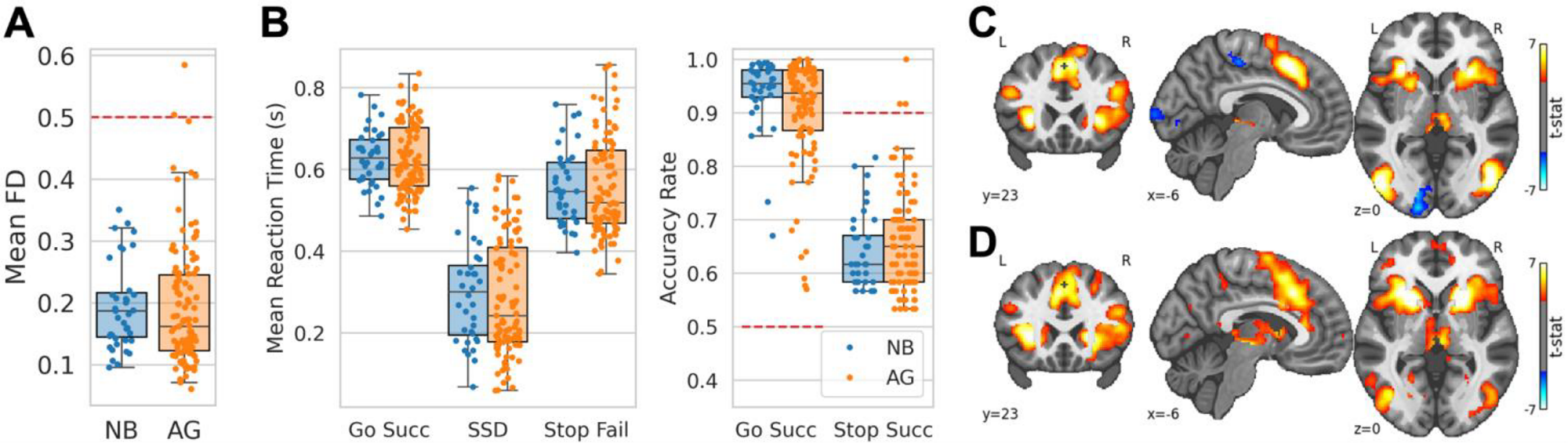
Summary of the SST fMRI. (A) There were no significant group differences in mean frame-wise displacement (FD) before outlier exclusion (*p *= 0.75; *n *= 137). Red horizontal dotted line indicates a mean FD of 0.5 mm; participants above this threshold were labeled as motion outliers. (B) Mean task response time and accuracy before excluding motion and performance outliers (*n *= 137). Red dotted lines indicate a go success rate threshold of 0.5 (performance below this threshold was excluded) and a stop success rate threshold of 0.9 (performance above this threshold was excluded). Detailed performance outlier criteria are provided in Supplementary Methods 1. (C) One-sample activation map of successful stop minus correct go contrast after excluding motion and performance outliers (*n *= 126) (D) One-sample activation map of unsuccessful stop minus correct go contrast (*n *= 126). The statistics were cluster corrected with a voxel-wise *p* < 0.001 and cluster α = 0.05. Abbreviations: FD: frame-wise displacement; NB: non-bereaved comparison group; AG: acute grief; SSD: stop signal delay.

One AG and two NB participants did not complete the SST fMRI. An additional two AG and one NB participants were excluded due to technical issues with response recording. The SST task data for the remaining 137 participants had a mean tSNR of 44.68 ± 8.76 (Supplementary Fig. S2C & S2D) and a mean FD of 0.21 ± 0.11 mm. There were no significant differences between groups for mean tSNR (AG: 44.97 ± 9.30; NB: 43.87 ± 7.07; *p* = 0.47) or mean FD (AG: 0.21 ± 0.12 mm; NB: 0.20 ± 0.07 mm; *p* = 0.75) (Fig. 6A). Two AG participants exhibited excessive head motion during the scan. Using the age-adjusted performance flags (see Section 2.2.2.2. Task fMRI and Supplementary Methods 1), seven AG and two NB participants were excluded. Mean response times and accuracy rates for the remaining 126 participants were within expected ranges and were statistically comparable between groups across conditions (*p* > 0.05; Fig. 6B).

We generated successful (successful stop minus successful go) and unsuccessful (unsuccessful stop minus successful go) inhibition contrast maps (*n* = 126) (Fig. 6C). Task contrast maps revealed significant activations for the successful stop minus correct go contrast in the IFG, pre-SMA, basal ganglia, anterior insula, and superior temporal sulcus, consistent with prior reports (Aron & Poldrack, 2006; Zhang et al., 2017). Similarly, activation maps for the unsuccessful stop minus correct go contrast showed overlapping activation, with additional activation in the dorsal ACC, thalamus, and visual areas. See Supplementary Figure S3 for results obtained using the ABCD performance flags (*n* = 112).

### 3.3 Longitudinal data

The study is ongoing; as of August 2025, 86 AG and 33 NB participants completed the 12-month MRI visit (Fig. 2). A total of 15 participants (12 AG and 3 NB) ended the study prematurely due to the following reasons: lost to follow-up (*n* = 6), dropped out (*n* = 3), met exclusion criteria (*n* = 6). Eleven of the 15 participants finished at least through the 6-month visit. The current attrition is 11.2% (12.2% in the AG group), within the proposed 15% attrition rate.

## 4 Discussion

This project aims to identify functional brain measures of emotional dysregulation in bereaved older adults. Such measures, along with collected characterization instruments, may be useful in predicting pathological grief trajectories and prolonged grief persistence. Moreover, these biological measures could, in the future, serve as novel targets for prevention and treatment strategies in older adults with AG at risk for maladaptive grief responses and the development of PGD, thereby promoting resilience. By integrating across multiple levels of ER analysis (brain circuit, behavior, and self-report), this project is well aligned with the multidimensional framework of the NIMH RDoC negative valence system (loss) construct.

The study design offers close monitoring of AG participants during one of life’s most stressful periods. Multidimensional psychiatric, medical, neuropsychological, and psychosocial variables are assessed repeatedly over 12 months. The inclusion of longitudinal functional neuroimaging at baseline and after 12 months uniquely allows examination of ER-related brain function changes associated with both prolonged grief and depression trajectories, as well as grief-specific symptom evolution over time. Moreover, the study design permits testing of potential mediators and moderators of the brain–clinical relationships, including personality and attachment styles, early life adversity, psychiatric history, medical burden, cognitive status, age-related structural brain changes, and contextual factors such as nature of the loss, relationship with the deceased, and social factors.

Although not a primary focus of the present aims, attachment theory may provide an additional lens for interpreting individual variability in emotional reactivity and regulatory control following bereavement. Neurobiological models of attachment emphasize interactions between limbic reactivity and prefrontal control systems during experiences of social separation (Long et al., 2020). Exploratory analyses will examine whether attachment dimensions, as measured by the SAAM, contribute to variability in ER-related neural and clinical trajectories over time.

Image quality control metrics, including tSNR and mean FD, are comparable with those reported in other multiband fMRI datasets (Hagler et al., 2019; Seok et al., 2020). Both the face–shape matching and SST paradigms demonstrate high accuracy rates, supporting data reliability. Despite extensive data collection across multiple visits (up to nine per completer), high participant retention and positive feedback indicate that study burden is acceptable.

Due to the COVID-19 pandemic, all procedures followed CDC and institutional guidelines, including physical distancing and use of personal protective equipment. Although routine COVID-19 screening was later discontinued, additional precautions were maintained given the older age of our study population. Daily visit volume was limited to allow adequate sanitization.

Overall study risk was minimal; however, rare emotional discomfort and distress arose during interviews, particularly among participants with elevated prolonged grief symptoms. Distress was mitigated by offering breaks or rescheduling assessments. Participants reporting significant discomfort were evaluated by a study physician to determine the need for medical or psychiatric care, with after-hours on-call faculty available as needed. Suicide risk monitoring procedures are detailed in Supplementary Methods 3, and participants had access to geriatric psychiatry and grief clinics. Acute medical concerns identified during study visits were referred to urgent care or the emergency department. Finally, all structural MRI scans were reviewed by a clinical neuroradiologist, with urgent and non-urgent findings communicated to the principal investigator in a timely manner.

### 4.1 Limitations

As an observational study, it is neither feasible nor ethical to completely avoid treatment-related variability. Therefore, current and past psychotropic medication use is not an exclusion criterion; a minority of AG participants are currently on antidepressant therapy (see Table 3). Stable antidepressant or low-dose benzodiazepines use is permitted if doses remain stable for at least 4 weeks prior to each MRI session. Because psychotropic medications may alter neuroimaging correlates of psychopathology, planned analyses will explore the moderating effects of antidepressants. Similarly, due to the modest sample size, formal subgroup analyses examining the potential confounding effects of prior grief experiences, medication status, medical illness burden, and relationship with the deceased will be limited. However, these variables may be considered as potential covariates and/or moderators where appropriate; any exploratory analyses will be interpreted with caution.

Without a non-bereaved depressed comparison group, conclusions regarding distinct ER circuit dysfunction in PGD versus LLD cannot be made. However, baseline depressive symptoms will be included as a covariate in longitudinal models and explored as a potential moderator. Findings will, therefore, be interpreted cautiously, with reference to existing meta-analyses and the neuroimaging literature on LLD, to contextualize group differences and avoid overattributing neural effects to grief-specific processes.

Another limitation relates to diagnostic evolution. Although PGD was formally recognized in the DSM-5-TR in Spring 2022, study recruitment began in 2020. Accordingly, AG participants who surpassed 1-year post-loss were initially evaluated using the diagnostic criteria available at study inception (i.e., using SCI-CG). Additionally, the Structured Clinical Interview for Prolonged Grief (SCIP), which aligns with the DSM-5-TR criteria, was incorporated into the protocol in February 2023.

This study does not include a direct self-report measure of ER (e.g., Emotion Regulation Questionnaire) or fMRI paradigms probing explicit ER strategies such as cognitive reappraisal or suppression. The selected paradigms capture ER-relevant neurocognitive components (i.e., affective reactivity and domain-general inhibitory control) but do not directly instantiate explicit ER strategies. Findings should, therefore, be interpreted as reflecting ER-relevant neural substrates rather than explicit ER processes per se; results from this investigation may inform the design of future studies that more comprehensively assess broader ER construct dysfunction.

Finally, while the present study focuses on ER as a primary neurobiological construct, our broader model of PGD (Goveas et al., 2026) integrates other transdiagnostic constructs, including reward processing and executive function (cognitive control, attention, working memory), which map onto large-scale networks and related neural circuitry (Blair et al., 2025; Goveas et al., 2026). Future work should test this model to more comprehensively assess the neurobiological underpinnings of PGD pathogenesis. Although issues have been raised regarding aspects of the ABCD SST paradigm (Bissett et al., 2021), the updated performance criteria steps outlined by Garavan et al. (2022) were implemented to ensure reliable measures of inhibitory control and associated brain function. While these stringent criteria may necessitate excluding some participants, appropriate statistical approaches will be applied to minimize bias and preserve power.

### 4.2 Conclusions

This study provides a comprehensive, multimodal framework for elucidating the neurobiological underpinnings of grief in older adults. This work represents a critical step toward bridging mechanistic neuroscience and clinical intervention science by identifying brain-based measures that may guide early detection, personalize therapeutic strategies, and monitor treatment response trajectory. Establishing reliable biomarkers of PGD vulnerability could ultimately transform approaches for prevention and management of PGD, particularly in late life, when bereavement is both prevalent and consequential for mental and physical health.

## Supplementary Material

Supplementary Material

## Data Availability

The 12-month longitudinal neuroimaging and clinical data described in the current manuscript are available upon reasonable request. Codes related to future data analysis will be made available via the DREAM (Developing Resilience to Ease Anguish in Mourning) Research GitHub repository (https://github.com/organizations/DREAM-research).

## References

[IMAG.a.1271-b1] 2013. Diagnostic and statistical manual of mental disorders, fifth edition (DSM-5) ed. American Psychiatric Association, Arlington. 10.1176/appi.books.9780890423349

[IMAG.a.1271-b2] Aizenstein, H. J., Andreescu, C., Edelman, K. L., Cochran, J. L., Price, J., Butters, M. A., Karp, J., Patel, M., & Reynolds, C. F., 3rd (2011). fMRI correlates of white matter hyperintensities in late-life depression. Am J Psychiatry, 168, 1075–1082. 10.1176/appi.ajp.2011.10060853 21799066 PMC3656408

[IMAG.a.1271-b3] Antonucci, T. C., Akiyama, H., & Takahashi, K. (2004). Attachment and close relationships across the life span. Attach Hum Dev, 6, 353–370. 10.1080/1461673042000303136 15764124

[IMAG.a.1271-b4] Arizmendi, B., Kaszniak, A. W., & O’Connor, M. F. (2016). Disrupted prefrontal activity during emotion processing in complicated grief: An fMRI investigation. Neuroimage, 124, 968–976. 10.1016/j.neuroimage.2015.09.054 26434802 PMC4668712

[IMAG.a.1271-b5] Aron, A. R., & Poldrack, R. A. (2006). Cortical and subcortical contributions to Stop signal response inhibition: Role of the subthalamic nucleus. J Neurosci, 26, 2424–2433. 10.1523/jneurosci.4682-05.2006 16510720 PMC6793670

[IMAG.a.1271-b6] Bastien, C. H., Vallieres, A., & Morin, C. M. (2001). Validation of the Insomnia Severity Index as an outcome measure for insomnia research. Sleep Med, 2, 297–307. 10.1016/s1389-9457(00)00065-4 11438246

[IMAG.a.1271-b7] Baumert, J., Simon, H., Gundel, H., Schmitt, C., & Ladwig, K. H. (2004). The impact of event scale—revised: Evaluation of the subscales and correlations to psychophysiological startle response patterns in survivors of a life-threatening cardiac event: An analysis of 129 patients with an implanted cardioverter defibrillator. J Affect Disord, 82, 29–41. 10.1016/j.jad.2003.09.006 15465574

[IMAG.a.1271-b8] Behzadi, Y., Restom, K., Liau, J., & Liu, T. T. (2007). A component based noise correction method (CompCor) for BOLD and perfusion based fMRI. Neuroimage, 37, 90–101. 10.1016/j.neuroimage.2007.04.042 17560126 PMC2214855

[IMAG.a.1271-b9] Bernstein, D. P., Ahluvalia, T., Pogge, D., & Handelsman, L. (1997). Validity of the Childhood Trauma Questionnaire in an adolescent psychiatric population. J Am Acad Child Adolesc Psychiatry, 36, 340–348. 10.1097/00004583-199703000-00012 9055514

[IMAG.a.1271-b10] Bernstein, D. P., Stein, J. A., Newcomb, M. D., Walker, E., Pogge, D., Ahluvalia, T., Stokes, J., Handelsman, L., Medrano, M., Desmond, D., & Zule, W. (2003). Development and validation of a brief screening version of the Childhood Trauma Questionnaire. Child Abuse Negl, 27, 169–190. 10.1016/s0145-2134(02)00541-0 12615092

[IMAG.a.1271-b11] Birn, R. M., Molloy, E. K., Patriat, R., Parker, T., Meier, T. B., Kirk, G. R., Nair, V. A., Meyerand, M. E., & Prabhakaran, V. (2013). The effect of scan length on the reliability of resting-state fMRI connectivity estimates. Neuroimage, 83, 550–558. 10.1016/j.neuroimage.2013.05.099 23747458 PMC4104183

[IMAG.a.1271-b12] Bissett, P. G., Hagen, M. P., Jones, H. M., & Poldrack, R. A. (2021). Design issues and solutions for stop-signal data from the Adolescent Brain Cognitive Development (ABCD) study. Elife, 10, e60185. 10.7554/elife.60185 33661097 PMC7997655

[IMAG.a.1271-b13] Blair, N. P., Hwang, G., Ward, B. D., Claesges, S. A., Webber, A. R., Hainsworth, K. R., Wang, Y., Reynolds, C. F., 3rd, Stein, E. A., & Goveas, J. S. (2025). Disrupted large-scale brain network connectivity in prolonged grief disorder: Relationship with grief-related avoidance, yearning, and intrusive thoughts. Biol Psychiatry Cogn Neurosci Neuroimaging. 10.1016/j.bpsc.2025.07.011 PMC12346161 40769298

[IMAG.a.1271-b14] Blevins, C. A., Weathers, F. W., Davis, M. T., Witte, T. K., & Domino, J. L. (2015). The posttraumatic stress disorder checklist for DSM-5 (PCL-5): Development and initial psychometric evaluation. J Trauma Stress, 28, 489–498. 10.1002/jts.22059 26606250

[IMAG.a.1271-b15] Bovin, M. J., Marx, B. P., Weathers, F. W., Gallagher, M. W., Rodriguez, P., Schnurr, P. P., & Keane, T. M. (2016). Psychometric properties of the PTSD Checklist for Diagnostic and Statistical Manual of Mental Disorders-Fifth Edition (PCL-5) in veterans. Psychol Assess, 28, 1379–1391. 10.1037/pas0000254 26653052

[IMAG.a.1271-b16] Brassen, S., Kalisch, R., Weber-Fahr, W., Braus, D. F., & Buchel, C. (2008). Ventromedial prefrontal cortex processing during emotional evaluation in late-life depression: A longitudinal functional magnetic resonance imaging study. Biol Psychiatry, 64, 349–355. 10.1016/j.biopsych.2008.03.022 18440493

[IMAG.a.1271-b17] Bryant, R. A., Andrew, E., & Korgaonkar, M. S. (2021). Distinct neural mechanisms of emotional processing in prolonged grief disorder. Psychol Med, 51, 587–595. 10.1017/s0033291719003507 31907095

[IMAG.a.1271-b18] Butters, N., Granholm, E., Salmon, D. P., Grant, I., & Wolfe, J. (1987). Episodic and semantic memory: A comparison of amnesic and demented patients. J Clin Exp Neuropsychol, 9, 479–497. 10.1080/01688638708410764 2959682

[IMAG.a.1271-b19] Casey, B. J., Cannonier, T., Conley, M. I., Cohen, A. O., Barch, D. M., Heitzeg, M. M., Soules, M. E., Teslovich, T., Dellarco, D. V., Garavan, H., Orr, C. A., Wager, T. D., Banich, M. T., Speer, N. K., Sutherland, M. T., Riedel, M. C., Dick, A. S., Bjork, J. M., Thomas, K. M., … Workgroup, A. I. A. (2018). The Adolescent Brain Cognitive Development (ABCD) study: Imaging acquisition across 21 sites. Dev Cogn Neurosci, 32, 43–54. 10.1016/j.dcn.2018.03.001 29567376 PMC5999559

[IMAG.a.1271-b20] Chen, G., Ward, B. D., Claesges, S. A., Li, S. J., & Goveas, J. S. (2020). Amygdala functional connectivity features in grief: A pilot longitudinal study. Am J Geriatr Psychiatry, 28, 1089–1101. 10.1016/j.jagp.2020.02.014 32253102 PMC7483593

[IMAG.a.1271-b21] Dhingra, I., Zhang, S., Zhornitsky, S., Le, T. M., Wang, W., Chao, H. H., Levy, I., & Li, C. R. (2020). The effects of age on reward magnitude processing in the monetary incentive delay task. Neuroimage, 207, 116368. 10.1016/j.neuroimage.2019.116368 31743790 PMC7463276

[IMAG.a.1271-b23] Eisma, M. C., & Stroebe, M. S. (2021). Emotion regulatory strategies in complicated grief: A systematic review. Behav Ther, 52, 234–249. 10.1016/j.beth.2020.04.004 33483120

[IMAG.a.1271-b24] Esteban, O., Markiewicz, C. J., Blair, R. W., Moodie, C. A., Isik, A. I., Erramuzpe, A., Kent, J. D., Goncalves, M., DuPre, E., Snyder, M., Oya, H., Ghosh, S. S., Wright, J., Durnez, J., Poldrack, R. A., & Gorgolewski, K. J. (2019). fMRIPrep: A robust preprocessing pipeline for functional MRI. Nat Methods, 16, 111–116. 10.1038/s41592-018-0235-4 30532080 PMC6319393

[IMAG.a.1271-b25] Fernandez-Alcantara, M., Verdejo-Roman, J., Cruz-Quintana, F., Perez-Garcia, M., Catena-Martinez, A., Fernandez-Avalos, M. I., & Perez-Marfil, M. N. (2020). Increased amygdala activations during the emotional experience of death-related pictures in complicated grief: An fMRI study. J Clin Med, 9, 851. 10.3390/jcm9030851 32245009 PMC7141501

[IMAG.a.1271-b26] Fischl, B., Salat, D. H., Busa, E., Albert, M., Dieterich, M., Haselgrove, C., van der Kouwe, A., Killiany, R., Kennedy, D., Klaveness, S., Montillo, A., Makris, N., Rosen, B., & Dale, A. M. (2002). Whole brain segmentation: Automated labeling of neuroanatomical structures in the human brain. Neuron, 33, 341–355. 10.1016/s0896-6273(02)00569-x 11832223

[IMAG.a.1271-b27] Fonov, V., Evans, A. C., Botteron, K., Almli, C. R., McKinstry, R. C., & Collins, D. L., Brain Development Cooperative, G. (2011). Unbiased average age-appropriate atlases for pediatric studies. Neuroimage, 54, 313–327. 10.1016/j.neuroimage.2010.07.033 20656036 PMC2962759

[IMAG.a.1271-b28] Freed, P. J., Yanagihara, T. K., Hirsch, J., & Mann, J. J. (2009). Neural mechanisms of grief regulation. Biol Psychiatry, 66, 33–40. 10.1016/j.biopsych.2009.01.019 19249748 PMC2782609

[IMAG.a.1271-b29] Freedman, M., Leach, L., Kaplan, E., Winocur, G., Shulman, K. I., & Delis, D. C. (1994). Clock drawing: A neuropsychological analysis. Oxford University Press, New York, NY. 10.1017/s0714980800013398

[IMAG.a.1271-b30] Garavan, H., Chaarani, B., Hahn, S., Allgaier, N., Juliano, A., Yuan, D. K., Orr, C., Watts, R., Wager, T. D., Ruiz de Leon, O., Hagler, D. J., Jr., & Potter, A. (2022). The ABCD stop signal data: Response to Bissett et al. Dev Cogn Neurosci, 57, 101144. 10.1016/j.dcn.2022.101144 35987133 PMC9411576

[IMAG.a.1271-b31] Gillath, O. Hart, J., Noftle, E. E., & Stockdale, G. D. (2009). Development and validation of a state adult attachment measure (SAAM). J Res Personal, 43, 362–373. 10.1016/j.jrp.2008.12.009

[IMAG.a.1271-b32] Golden, C. J. (1978). The stroop color and word test (Manual), Stoetling, Chicago. 10.1037/t06065-000

[IMAG.a.1271-b33] Goldin, P. R., Hutcherson, C. A., Ochsner, K. N., Glover, G. H., Gabrieli, J. D., & Gross, J. J. (2005). The neural bases of amusement and sadness: A comparison of block contrast and subject-specific emotion intensity regression approaches. Neuroimage, 27, 26–36. 10.1016/j.neuroimage.2005.03.018 15890534

[IMAG.a.1271-b34] Goveas, J. S., Hwang, G., Blair, N. P., Stein, E. A., & Reynolds III, C.F. (2026). Prolonged grief disorder in later life: Advancing our understanding of biopsychosocial mechanisms to guide future personalized interventions. Neuropsychopharmacology. In Press. 10.1038/s41386-026-02329-x PMC13005943 41577994

[IMAG.a.1271-b35] Gunning-Dixon, F. M., Gur, R. C., Perkins, A. C., Schroeder, L., Turner, T., Turetsky, B. I., Chan, R. M., Loughead, J. W., Alsop, D. C., Maldjian, J., & Gur, R. E. (2003). Age-related differences in brain activation during emotional face processing. Neurobiol Aging, 24, 285–295. 10.1016/s0197-4580(02)00099-4 12498962

[IMAG.a.1271-b36] Gurrentz, B., & Mayol-Garcia, Y. (2021). Marriage, divorce, widowhood remain prevalent among older populations. United States Census Bureau. 10.1093/oso/9780190237578.003.0004

[IMAG.a.1271-b37] Hagler, D. J., Jr., Hatton, S., Cornejo, M. D., Makowski, C., Fair, D. A., Dick, A. S., Sutherland, M. T., Casey, B. J., Barch, D. M., Harms, M. P., Watts, R., Bjork, J. M., Garavan, H. P., Hilmer, L., Pung, C. J., Sicat, C. S., Kuperman, J., Bartsch, H., Xue, F., … Dale, A. M. (2019). Image processing and analysis methods for the Adolescent Brain Cognitive Development Study. Neuroimage, 202, 116091. 10.1016/j.neuroimage.2019.116091 31415884 PMC6981278

[IMAG.a.1271-b38] Hamilton, M. (1959). The assessment of anxiety states by rating. Br J Med Psychol, 32, 50–55. 10.1111/j.2044-8341.1959.tb00467.x 13638508

[IMAG.a.1271-b39] Hamilton, M. (1980). Rating depressive patients. J Clin Psychiatry, 41, 21–24. 10.1016/0006-3223(96)84032-7 7440521

[IMAG.a.1271-b40] Hariri, A. R., Tessitore, A., Mattay, V. S., Fera, F., & Weinberger, D. R. (2002). The amygdala response to emotional stimuli: A comparison of faces and scenes. Neuroimage, 17, 317–323. 10.1006/nimg.2002.1179 12482086

[IMAG.a.1271-b41] Hoffmann, B. M., Blair, N. P., McAuliffe, T. L., Hwang, G., Larson, E., Claesges, S. A., Webber, A., Reynolds, C. F., 3rd, & Goveas, J. S. (2024). Neuropsychological correlates of early grief in bereaved older adults. Int Psychogeriatr, 36, 1064–1069. 10.1017/s1041610224000048 38462965 PMC11387951

[IMAG.a.1271-b42] Hwang, G., Blair, N. P., Ward, B. D., McAuliffe, T. L., Claesges, S. A., Webber, A. R., Hainsworth, K. R., Wang, Y., Reynolds, C. F., Stein, E. A., & Goveas, J. S. (2024). Amygdala-centered emotional processing in prolonged grief disorder: Relationship with clinical symptomatology. Biol Psychiatry Cogn Neurosci Neuroimaging, 10, 1284–1293. 10.1016/j.bpsc.2024.12.008 39725082 PMC12185774

[IMAG.a.1271-b43] Ito, M., Nakajima, S., Fujisawa, D., Miyashita, M., Kim, Y., Shear, M. K., Ghesquiere, A., & Wall, M. M. (2012). Brief measure for screening complicated grief: Reliability and discriminant validity. PLoS One, 7, e31209. 10.1371/journal.pone.0031209 22348057 PMC3279351

[IMAG.a.1271-b44] Jenkinson, M., Beckmann, C. F., Behrens, T. E., Woolrich, M. W., & Smith, S. M. (2012). FSL. Neuroimage, 62, 782–790. 10.1016/j.neuroimage.2011.09.015 21979382

[IMAG.a.1271-b45] Jeste, D. V., Palmer, B. W., Appelbaum, P. S., Golshan, S., Glorioso, D., Dunn, L. B., Kim, K., Meeks, T., & Kraemer, H. C. (2007). A new brief instrument for assessing decisional capacity for clinical research. Arch Gen Psychiatry, 64, 966–974. 10.1001/archpsyc.64.8.966 17679641

[IMAG.a.1271-b46] John, O. P., & Srivastava, S. (1999). The Big-Five trait taxonomy: History, measurement, and theoretical perspectives. Guilford Press, New York. 10.1037/e302372005-016

[IMAG.a.1271-b47] Kaplan, E. F., Goodglass, H., & Weintraub, S. (1983). Boston naming test. Lea & Febiger, Philadelphia, P.A. 10.1037/t27208-000

[IMAG.a.1271-b48] Kim, H., Zhu, X., Zhao, Y., Bell, S. A., Gehrman, P. R., Cohen, D., Devanand, D. P., Goldberg, T. E., Lee, S., & Alzheimer’s Disease Neuroimaging, I. (2023). Resting-state functional connectivity changes in older adults with sleep disturbance and the role of amyloid burden. Mol Psychiatry, 28, 4399–4406. 10.1038/s41380-023-02214-9 37596355 PMC10842478

[IMAG.a.1271-b49] Koenig, H. G., Westlund, R. E., George, L. K., Hughes, D. C., Blazer, D. G., & Hybels, C. (1993). Abbreviating the Duke Social Support Index for use in chronically ill elderly individuals. Psychosomatics, 34, 61–69. 10.1016/s0033-3182(93)71928-3 8426892

[IMAG.a.1271-b50] Kroenke, K., Spitzer, R. L., & Williams, J. B. (2001). The PHQ-9: Validity of a brief depression severity measure. J Gen Intern Med, 16, 606–613. 10.1046/j.1525-1497.2001.016009606.x 11556941 PMC1495268

[IMAG.a.1271-b51] Kurtzer, G. M., Sochat, V., & Bauer, M. W. (2017). Singularity: Scientific containers for mobility of compute. PLoS One, 12, e0177459. 10.1371/journal.pone.0177459 28494014 PMC5426675

[IMAG.a.1271-b52] Lawton, M. P., & Brody, E. M. (1969). Assessment of older people: Self-maintaining and instrumental activities of daily living. Gerontologist, 9, 179–186. 10.1093/geront/9.3_part_1.179 5349366

[IMAG.a.1271-b53] Lemche, E., Giampietro, V. P., Surguladze, S. A., Amaro, E. J., Andrew, C. M., Williams, S. C., Brammer, M. J., Lawrence, N., Maier, M. A., Russell, T. A., Simmons, A., Ecker, C., Joraschky, P., & Phillips, M. L. (2006). Human attachment security is mediated by the amygdala: Evidence from combined fMRI and psychophysiological measures. Hum Brain Mapp, 27, 623–635. 10.1002/hbm.20206 16284946 PMC6871466

[IMAG.a.1271-b54] Li, W., Ward, B. D., Xie, C., Jones, J. L., Antuono, P. G., Li, S. J., & Goveas, J. S. (2015). Amygdala network dysfunction in late-life depression phenotypes: Relationships with symptom dimensions. J Psychiatr Res, 70, 121–129. 10.1016/j.jpsychires.2015.09.002 26424431 PMC4605880

[IMAG.a.1271-b55] Lobb, E. A., Kristjanson, L. J., Aoun, S. M., Monterosso, L., Halkett, G. K., & Davies, A. (2010). Predictors of complicated grief: A systematic review of empirical studies. Death Stud, 34, 673–698. 10.1080/07481187.2010.496686 24482845

[IMAG.a.1271-b104] Long, M., Verbeke, W., Ein-Dor, T., & Vrtička, P. (2020). A functional neuro-anatomical model of human attachment (*NAMA*): Insights from first- and second-person social neuroscience. Cortex, 126, 281–321. 10.1016/j.cortex.2020.01.010 32092496

[IMAG.a.1271-b56] Lubben, J., Blozik, E., Gillmann, G., Iliffe, S., von Renteln Kruse, W., Beck, J. C., & Stuck, A. E. (2006). Performance of an abbreviated version of the Lubben Social Network Scale among three European community-dwelling older adult populations. Gerontologist, 46, 503–513. 10.1093/geront/46.4.503 16921004

[IMAG.a.1271-b57] Lucas, J. A., Ivnik, R. J., Smith, G. E., Bohac, D. L., Tangalos, E. G., Kokmen, E., Graff-Radford, N. R., & Petersen, R. C. (1998). Normative data for the Mattis Dementia Rating Scale. J Clin Exp Neuropsychol, 20, 536–547. 10.1076/jcen.20.4.536.1469 9892057

[IMAG.a.1271-b58] Maccallum, F., & Bryant, R. A. (2013). A Cognitive Attachment Model of prolonged grief: Integrating attachments, memory, and identity. Clin Psychol Rev, 33, 713–727. 10.1016/j.cpr.2013.05.001 23792468

[IMAG.a.1271-b59] McConnell, M. H., Killgore, W. D. S., & O’Connor, M. F. (2018). Yearning predicts subgenual anterior cingulate activity in bereaved individuals. Heliyon, 4, e00852. 10.1016/j.heliyon.2018.e00852 30364703 PMC6197542

[IMAG.a.1271-b60] Miller, M. D., Paradis, C. F., Houck, P. R., Mazumdar, S., Stack, J. A., Rifai, A. H., Mulsant, B., & Reynolds, C. F., 3rd (1992). Rating chronic medical illness burden in geropsychiatric practice and research: Application of the Cumulative Illness Rating Scale. Psychiatry Res, 41, 237–248. 10.1016/0165-1781(92)90005-n 1594710

[IMAG.a.1271-b61] Naismith, S. L., Norrie, L. M., Mowszowski, L., & Hickie, I. B. (2012). The neurobiology of depression in later-life: Clinical, neuropsychological, neuroimaging and pathophysiological features. Prog Neurobiol, 98, 99–143. 10.1016/j.pneurobio.2012.05.009 22609700

[IMAG.a.1271-b62] Nasreddine, Z. S., Phillips, N., & Chertkow, H. (2012). Normative data for the Montreal Cognitive Assessment (MoCA) in a population-based sample. Neurology, 78, 765–766; author reply 766. 10.1212/01.wnl.0000413072.54070.a3 22391608

[IMAG.a.1271-b63] Nasreddine, Z. S., Phillips, N. A., Bedirian, V., Charbonneau, S., Whitehead, V., Collin, I., Cummings, J. L., & Chertkow, H. (2005). The Montreal Cognitive Assessment, MoCA: A brief screening tool for mild cognitive impairment. J Am Geriatr Soc, 53, 695–699. 10.1111/j.1532-5415.2005.53221.x 15817019

[IMAG.a.1271-b64] Nelson, H. E. (1982). Nelson adult reading test. The National Hospital for Nervous Disease, London. 10.1037/t51600-000

[IMAG.a.1271-b65] O’Connor, M. F. (2012). Immunological and neuroimaging biomarkers of complicated grief. Dialogues Clin Neurosci, 14, 141–148. 10.31887/dcns.2012.14.2/mfoconnor 22754286 PMC3384442

[IMAG.a.1271-b66] O’Connor, M. F., & Sussman, T. J. (2014). Developing the yearning in situations of loss scale: Convergent and discriminant validity for bereavement, romantic breakup, and homesickness. Death Stud, 38, 450–458. 10.1080/07481187.2013.782928 24758215

[IMAG.a.1271-b67] O’Connor, M. F., Wellisch, D. K., Stanton, A. L., Eisenberger, N. I., Irwin, M. R., & Lieberman, M. D. (2008). Craving love? Enduring grief activates brain’s reward center. Neuroimage, 42, 969–972. 10.1016/j.neuroimage.2008.04.256 18559294 PMC2553561

[IMAG.a.1271-b68] Perez, H. C. S., Ikram, M. A., Direk, N., & Tiemeier, H. (2018). Prolonged grief and cognitive decline: A prospective population-based study in middle-aged and older persons. Am J Geriatr Psychiatry, 26, 451–460. 10.1016/j.jagp.2017.12.003 29329723

[IMAG.a.1271-b69] Phelps, E. A. (2006). Emotion and cognition: Insights from studies of the human amygdala. Annu Rev Psychol, 57, 27–53. 10.1146/annurev.psych.56.091103.070234 16318588

[IMAG.a.1271-b70] Posner, K., Brown, G. K., Stanley, B., Brent, D. A., Yershova, K. V., Oquendo, M. A., Currier, G. W., Melvin, G. A., Greenhill, L., Shen, S., & Mann, J. J. (2011). The Columbia-Suicide Severity Rating Scale: Initial validity and internal consistency findings from three multisite studies with adolescents and adults. Am J Psychiatry, 168, 1266–1277. 10.1176/appi.ajp.2011.10111704 22193671 PMC3893686

[IMAG.a.1271-b71] Power, J. D., Barnes, K. A., Snyder, A. Z., Schlaggar, B. L., & Petersen, S. E. (2012). Spurious but systematic correlations in functional connectivity MRI networks arise from subject motion. Neuroimage, 59, 2142–2154. 10.1016/j.neuroimage.2011.10.018 22019881 PMC3254728

[IMAG.a.1271-b72] Prigerson, H. G., Bierhals, A. J., Kasl, S. V., Reynolds, C. F., 3rd, Shear, M. K., Day, N., Beery, L. C., Newsom, J. T., & Jacobs, S. (1997). Traumatic grief as a risk factor for mental and physical morbidity. Am J Psychiatry, 154, 616–623. 10.1176/ajp.154.5.616 9137115

[IMAG.a.1271-b73] Prigerson, H. G., Boelen, P. A., Xu, J. H., Smith, K. V., & Maciejewski, P. K. (2021). Validation of the new DSM-5-TR criteria for prolonged grief disorder and the PG-13-Revised (PG-13-R) scale. World Psychiatry, 20, 96–106. 10.1002/wps.20823 33432758 PMC7801836

[IMAG.a.1271-b74] Prigerson, H. G., Frank, E., Kasl, S. V., Reynolds, C. F.,3rd, Anderson, B., Zubenko, G. S., Houck, P. R., George, C. J., & Kupfer, D. J. (1995). Complicated grief and bereavement-related depression as distinct disorders: Preliminary empirical validation in elderly bereaved spouses. Am J Psychiatry, 152, 22–30. 10.1176/ajp.152.1.22 7802116

[IMAG.a.1271-b75] Prigerson, H. G., Maciejewski, P. K., Reynolds, C. F. 3rd, Bierhals, A. J., Newsom, J. T., Fasiczka, A., Frank, E., Doman, J., & Miller, M. (1995). Inventory of complicated grief: A scale to measure maladaptive symptoms of loss. Psychiatry Res, 59, 65–79. 10.1016/0165-1781(95)02757-2 8771222

[IMAG.a.1271-b76] Prigerson, H. G., Shear, M. K., Newsom, J. T., Frank, E., Reynolds, C. F., 3rd, Maciejewski, P. K., Houck, P. R., Bierhals, A. J., & Kupfer, D. J. (1996). Anxiety among widowed elders: Is it distinct from depression and grief? Anxiety, 2, 1–12. 10.1002/(sici)1522-7154(1996)2:1<1::aid-anxi1>3.3.co;2-2 9160593

[IMAG.a.1271-b77] Prigerson, H. G., Shear, M. K., & Reynolds, C. F., 3rd (2022). Prolonged grief disorder diagnostic criteria-helping those with maladaptive grief responses. JAMA Psychiatry, 79, 277–278. 10.1001/jamapsychiatry.2021.4201 35107569

[IMAG.a.1271-b78] Prudic, J., Haskett, R. F., Mulsant, B., Malone, K. M., Pettinati, H. M., Stephens, S., Greenberg, R., Rifas, S. L., & Sackeim, H. A. (1996). Resistance to antidepressant medications and short-term clinical response to ECT. Am J Psychiatry, 153, 985–992. 10.1016/0924-977x(96)87393-7 8678194

[IMAG.a.1271-b79] Pruim, R. H. R., Mennes, M., van Rooij, D., Llera, A., Buitelaar, J. K., & Beckmann, C. F. (2015). ICA-AROMA: A robust ICA-based strategy for removing motion artifacts from fMRI data. Neuroimage, 112, 267–277. 10.1016/j.neuroimage.2015.02.064 25770991

[IMAG.a.1271-b80] Reitan, R. M. (1955). The relation of the trail making test to organic brain damage. J Consult Psychol, 19, 393–394. 10.1037/h0044509 13263471

[IMAG.a.1271-b81] Roy, A. K., Shehzad, Z., Margulies, D. S., Kelly, A. M., Uddin, L. Q., Gotimer, K., Biswal, B. B., Castellanos, F. X., & Milham, M. P. (2009). Functional connectivity of the human amygdala using resting state fMRI. Neuroimage, 45, 614–626. 10.1016/j.neuroimage.2008.11.030 19110061 PMC2735022

[IMAG.a.1271-b82] Russell, D. W. (1996). UCLA Loneliness Scale (Version 3): Reliability, validity, and factor structure. J Pers Assess, 66, 20–40. 10.1207/s15327752jpa6601_2 8576833

[IMAG.a.1271-b83] Sackeim, H. A. (2001). The definition and meaning of treatment-resistant depression. J Clin Psychiatry, 62 Suppl 16, 10–17. 10.1001/jamapsychiatry.2016.2586 11480879

[IMAG.a.1271-b84] Savage, H. S., Mulders, P. C. R., van Eijndhoven, P. F. P., van Oort, J., Tendolkar, I., Vrijsen, J. N., Beckmann, C. F., & Marquand, A. F. (2024). Dissecting task-based fMRI activity using normative modelling: An application to the Emotional Face Matching Task. Commun Biol, 7, 888. 10.1038/s42003-024-06573-z 39033247 PMC11271583

[IMAG.a.1271-b85] Seok, D., Smyk, N., Jaskir, M., Cook, P., Elliott, M., Girelli, T., Scott, J. C., Balderston, N., Beer, J., Stock, J., Makhoul, W., Gur, R. C., Davatzikos, C., Shinohara, R., & Sheline, Y. (2020). Dimensional connectomics of anxious misery, a human connectome study related to human disease: Overview of protocol and data quality. Neuroimage Clin, 28, 102489. 10.1016/j.nicl.2020.102489 33395980 PMC7708855

[IMAG.a.1271-b86] Shear, K., Monk, T., Houck, P., Melhem, N., Frank, E., Reynolds, C., & Sillowash, R. (2007). An attachment-based model of complicated grief including the role of avoidance. Eur Arch Psychiatry Clin Neurosci, 257, 453–461. 10.1007/s00406-007-0745-z 17629727 PMC2806638

[IMAG.a.1271-b87] Shear, K., & Shair, H. (2005). Attachment, loss, and complicated grief. Dev Psychobiol, 47, 253–267. 10.1002/dev.20091 16252293

[IMAG.a.1271-b88] Shear, M. K., Reynolds, C. F., 3rd, Simon, N. M., Zisook, S., Wang, Y., Mauro, C., Duan, N., Lebowitz, B., & Skritskaya, N. (2016). Optimizing treatment of complicated grief: A randomized clinical trial. JAMA Psychiatry, 73, 685–694. 10.1001/jamapsychiatry.2016.0892 27276373 PMC5735848

[IMAG.a.1271-b89] Shear, M. K., Wang, Y., Skritskaya, N., Duan, N., Mauro, C., & Ghesquiere, A. (2014). Treatment of complicated grief in elderly persons: A randomized clinical trial. JAMA Psychiatry, 71, 1287–1295. 10.1001/jamapsychiatry.2014.1242 25250737 PMC5705174

[IMAG.a.1271-b90] St Jacques, P. L., Bessette-Symons, B., & Cabeza, R. (2009). Functional neuroimaging studies of aging and emotion: Fronto-amygdalar differences during emotional perception and episodic memory. J Int Neuropsychol Soc, 15, 819–825. 10.1017/s1355617709990439 19703320 PMC3633489

[IMAG.a.1271-b91] Stroebe, M., Schut, H., & Stroebe, W. (2007). Health outcomes of bereavement. Lancet, 370, 1960–1973. 10.1016/s0140-6736(07)61816-9 18068517

[IMAG.a.1271-b92] Szanto, K., Prigerson, H., Houck, P., Ehrenpreis, L., & Reynolds, C. F., 3rd (1997). Suicidal ideation in elderly bereaved: The role of complicated grief. Suicide Life Threat Behav, 27, 194–207. 10.1111/j.1943-278x.1997.tb00291.x 9260302

[IMAG.a.1271-b93] Tadayonnejad, R., & Ajilore, O. (2014). Brain network dysfunction in late-life depression: A literature review. J Geriatr Psychiatry Neurol, 27, 5–12. 10.1177/0891988713516539 24381233

[IMAG.a.1271-b94] Tessitore, A., Hariri, A. R., Fera, F., Smith, W. G., Das, S., Weinberger, D. R., & Mattay, V. S. (2005). Functional changes in the activity of brain regions underlying emotion processing in the elderly. Psychiatry Res, 139, 9–18. 10.1016/j.pscychresns.2005.02.009 15936178

[IMAG.a.1271-b95] Tozzi, L., Staveland, B., Holt-Gosselin, B., Chesnut, M., Chang, S. E., Choi, D., Shiner, M., Wu, H., Lerma-Usabiaga, G., Sporns, O., Barch, D. M., Gotlib, I. H., Hastie, T. J., Kerr, A. B., Poldrack, R. A., Wandell, B. A., Wintermark, M., & Williams, L. M. (2020). The human connectome project for disordered emotional states: Protocol and rationale for a research domain criteria study of brain connectivity in young adult anxiety and depression. Neuroimage, 214, 116715. 10.1016/j.neuroimage.2020.116715 32147367 PMC8597395

[IMAG.a.1271-b96] Verbruggen, F., & Logan, G. D. (2008). Response inhibition in the stop-signal paradigm. Trends Cogn Sci, 12, 418–424. 10.1016/j.tics.2008.07.005 18799345 PMC2709177

[IMAG.a.1271-b97] Verdery, A. M., Smith-Greenaway, E., Margolis, R., & Daw, J. (2020). Tracking the reach of COVID-19 kin loss with a bereavement multiplier applied to the United States. Proc Natl Acad Sci U S A, 117, 17695–17701. 10.1073/pnas.2007476117 32651279 PMC7395491

[IMAG.a.1271-b98] Wang, L., McCarthy, G., Song, A. W., & Labar, K. S. (2005). Amygdala activation to sad pictures during high-field (4 tesla) functional magnetic resonance imaging. Emotion, 5, 12–22. 10.1037/1528-3542.5.1.12 15755216

[IMAG.a.1271-b99] Watson, D., Weber, K., Assenheimer, J. S., Clark, L. A., Strauss, M. E., & McCormick, R. A. (1995). Testing a tripartite model: I. Evaluating the convergent and discriminant validity of anxiety and depression symptom scales. J Abnorm Psychol, 104, 3–14. 10.1037/0021-843x.104.1.3 7897050

[IMAG.a.1271-b100] Wechsler, D. (1987). Manual for the Wechsler memory scale-revised. Psychological Corporation, San Antonio. 10.1016/0887-6177(88)90053-4

[IMAG.a.1271-b101] Wechsler, D. (2008). Wechsler adult intelligence scale - fourth edition (WAIS-IV). Psychological Corporation, Bloomington, MN. 10.53841/bpstest.2010.wais4

[IMAG.a.1271-b102] Xiao, T., Zhang, S., Lee, L. E., Chao, H. H., van Dyck, C., & Li, C. R. (2018). Exploring age-related changes in resting state functional connectivity of the amygdala: From young to middle adulthood. Front Aging Neurosci, 10, 209. 10.3389/fnagi.2018.00209 30061823 PMC6055042

[IMAG.a.1271-b103] Zhang, R., Geng, X., & Lee, T. M. C. (2017). Large-scale functional neural network correlates of response inhibition: An fMRI meta-analysis. Brain Struct Funct, 222, 3973–3990. 10.1007/s00429-017-1443-x 28551777 PMC5686258

