# Supplementary material for: Emotion regulation in prolonged grief disorder in later life: Protocol and rationale for a longitudinal neuroimaging study
Source: Imaging Neurosci (Camb). 2026 Jun 16;4:IMAG.a.1271. doi: 10.1162/IMAG.a.1271 (PMC13274567; doi:10.1162/IMAG.a.1271)
Supplement: Supplementary Material [file IMAG.a.1271_supp.pdf]

Online Supplementary Material for

"Emotion Regulation in Prolonged Grief Disorder in Later Life: Protocol and Rationale for a Longitudinal Neuroimaging Study"

Table of Contents

Figure S1. Multidisciplinary research team members and roles 2
Methods S1. SST task performance flags 3
Methods S2. Example fMRIPrep Boilerplate 4
Methods S3. Suicide risk monitoring procedure 7
Methods S4. MRI Quality Assurance 8
Figure S2. Summary of temporal signal-to-noise ratio (tSNR) from task fMRI 10
Figure S3. SST task activation using stringent performance flags 11
References 12

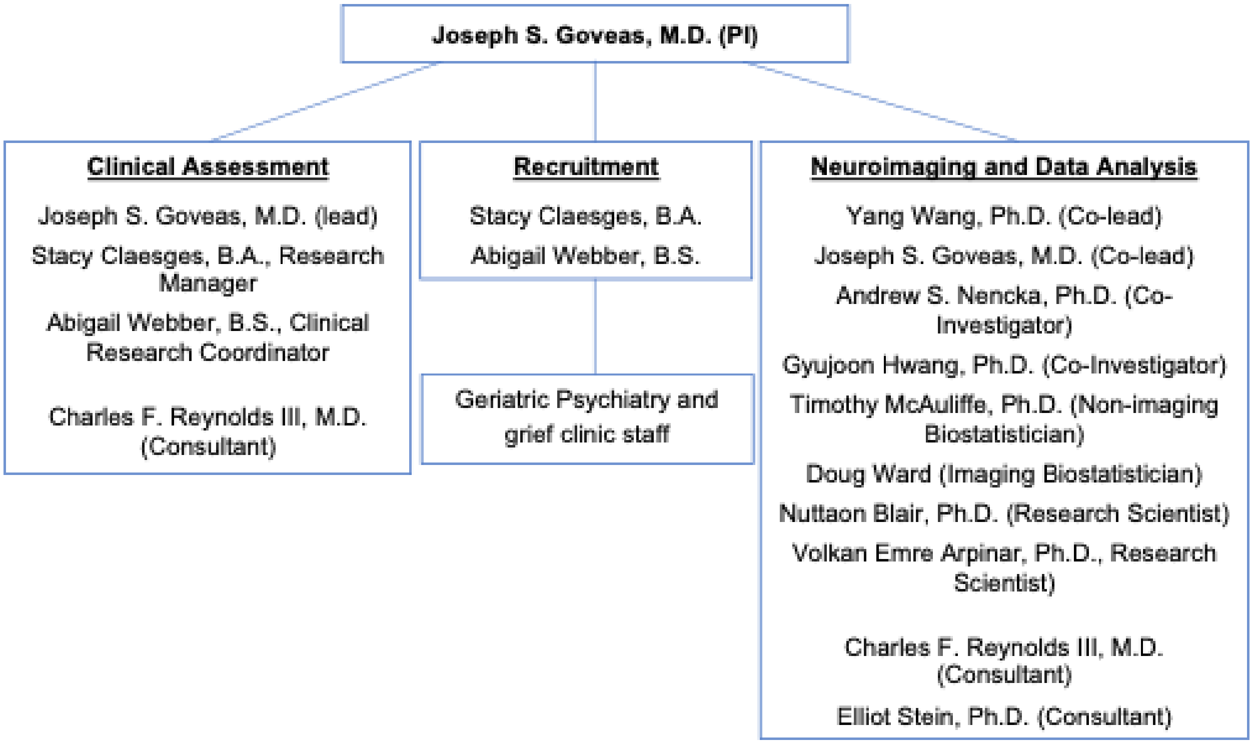

Figure S1. Multidisciplinary research team members and roles

Schematic representation of the administrative structure of the research study team. The study is led by the DREAM Lab (Developing Resilience to Ease Anguish in Mourning) at the Medical College of Wisconsin.

Methods S1. SST task performance flags

Concerns have been raised about response patterns suggesting inadequate task engagement, and several performance flags have been proposed to identify such cases (Garavan et al., 2022). Garavan et al. examined the Adolescent Brain Cognitive Development (ABCD) adolescent sample (n = 5,258) to determine which criteria conservatively identify poor task engagement, as such cases complicate interpretation of the task results. They proposed flagging cases with "Go" omission rate ≥30%, late "Go" rate ≥30%, incorrect "Go" rate ≥30%, correct "Go" rate ≤60%, correct "Go" response time faster than incorrect "Stop" response time, and "Stop" trial accuracy <20% or > 80%. Using these criteria, out of 135 participants with usable SST fMRI data, six participants were excluded for "Go" omission rate ≥30%, nine participants for correct "Go" response time faster than incorrect "Stop" response time, and eight participants for "Stop" trial accuracy <20% or >80%, leaving n = 112 for the subsequent analyses.

Given the older age of our sample, we developed a set of age-appropriate performance flags. We propose to flag response data with "Go" omission rate ≥40%, late "Go" rate ≥40%, incorrect "Go" rate ≥40%, correct "Go" rate ≤50%, a ≥50ms difference between correct "Go" and incorrect "Stop" response times, and "Stop" trial accuracy <20% or >90%. Additionally, we flagged participants with the estimated stop signal reaction time (SSRT) <50 ms. Using these criteria, out of the 135 participants, three participants were excluded for "Go" omission rate ≥40%, one for a ≥50 ms difference between correct "Go" and incorrect "Stop" response times, three for "Stop" trial accuracy <20% or >90%, and two for SSRT <50 ms, leaving n = 126 for the subsequent analyses.

For participants passing each set of criteria, we generated successful (successful stop minus successful go) and unsuccessful (unsuccessful stop minus successful go) inhibition contrast maps. Results from the full sample (***Figure 6C***) and the more stringently filtered sample (***Supplementary Figure 2***) yielded similar one-sample activation patterns.

Methods S2. Example fMRIPrep Boilerplate

The following is boilerplate text provided by fMRIPrep.

First, a reference volume and its skull-stripped version were generated using a custom methodology of *fMRIPrep*. Head-motion parameters with respect to the BOLD reference (transformation matrices, and six corresponding rotation and translation parameters) are estimated before any spatiotemporal filtering using `mcflirt` [FSL 6.0.5.1:57b01774, @mcflirt].

The estimated *fieldmap* was then aligned with rigid-registration to the target EPI (echo-planar imaging) reference run. The field coefficients were mapped on to the reference EPI using the transform. BOLD runs were slice-time corrected to 0s (0 of slice acquisition range 0s-0.72s) using `3dTshift` from AFNI [@afni, RRID:SCR_005927]. The BOLD reference was then co-registered to the T1w reference using `bbregister` (FreeSurfer) which implements boundary-based registration [@bbr]. Co-registration was configured with six degrees of freedom.

Several confounding time-series were calculated based on the *preprocessed BOLD*: framewise displacement (FD), DVARS and three region-wise global signals. FD was computed using two formulations following Power (absolute sum of relative motions, @power_fd_dvars) and Jenkinson (relative root mean square displacement between affines, @mcflirt). FD and DVARS are calculated for each functional run, both using their implementations in *Nipype* [following the definitions by @power_fd_dvars].

The three global signals are extracted within the CSF, the WM, and the whole-brain masks. Additionally, a set of physiological regressors were extracted to allow for component-based noise correction [*CompCor*, @compcor]. Principal components are estimated after high-pass filtering the *preprocessed BOLD* time-series (using a discrete cosine filter with 128s cut-off) for the two *CompCor* variants: temporal (tCompCor) and anatomical (aCompCor).

tCompCor components are then calculated from the top 2% variable voxels within the brain mask. For aCompCor, three probabilistic masks (CSF, WM and combined CSF+WM) are generated in anatomical space. The implementation differs from that of Behzadi et al. in that instead of eroding the masks by 2 pixels on BOLD space, a mask of pixels that likely contain a volume fraction of GM is subtracted from the aCompCor masks. This mask is obtained by dilating a GM mask extracted from the FreeSurfer's *aseg* segmentation, and it ensures components are not extracted from voxels containing a minimal fraction of GM. Finally, these masks are resampled into BOLD space and binarized by thresholding at 0.99 (as in the original implementation). Components are also calculated separately within the WM and CSF masks. For each CompCor decomposition, the *k* components with the largest singular values are retained, such that the retained components' time series are sufficient to explain 50 percent of variance across the nuisance mask (CSF, WM, combined, or temporal). The remaining components are dropped from consideration.

The head-motion estimates calculated in the correction step were also placed within the corresponding confounds file. The confound time series derived from head motion estimates and global signals were expanded with the inclusion of temporal derivatives and quadratic terms for each [@confounds_satterthwaite_2013]. Frames that exceeded a threshold of 0.5 mm FD or 1.5 standardized DVARS were annotated as motion outliers. Additional nuisance timeseries are calculated by means of principal components analysis of the signal found within a thin band (*crown*) of voxels around the edge of the brain, as proposed by [@patriat_improved_2017]. The BOLD time-series were resampled into standard space, generating a *preprocessed BOLD run in MNI152NLin6Asym space*. First, a reference volume and its skull-stripped version were generated using a custom methodology of *fMRIPrep*.

Automatic removal of motion artifacts using independent component analysis [ICA-AROMA, @aroma] was performed on the *preprocessed BOLD on MNI space* time-series after removal

of non-steady state volumes and spatial smoothing with an isotropic, Gaussian kernel of 6mm FWHM (full-width half-maximum). Corresponding "non-aggresively" denoised runs were produced after such smoothing. Additionally, the "aggressive" noise-regressors were collected and placed in the corresponding confounds file.

All resamplings can be performed with *a single interpolation step* by composing all the pertinent transformations (i.e. head-motion transform matrices, susceptibility distortion correction when available, and co-registrations to anatomical and output spaces). Gridded (volumetric) resamplings were performed using `antsApplyTransforms` (ANTs), configured with Lanczos interpolation to minimize the smoothing effects of other kernels [@lanczos]. Non-gridded (surface) resamplings were performed using `mri_vol2surf`(FreeSurfer).

Methods S3. Suicide risk monitoring procedure

Participants are monitored for suicidality at every visit and potential risks are addressed as below. 1) Mild risk (feels life is not worth living/wishes he/she was better off dead): Study physician will determine that there is no active suicide plan or intent. Steps to take if there are increases in suicide risk will be documented. 2) Moderate risk (active ideation but no specific plan): Once the participant confirms that there is no current intent to act on suicidal plans, study physician will determine the estimated level of risk and deterrents. A detailed description of safety plan and steps to take if suicide risk increases will be documented. 3) High risk: If an acute suicide or homicide risk (with a plan to harm self) is suspected at any point, the participant is accompanied by the study physician directly to the Emergency Department, and, if needed, emergency steps are taken to prevent a suicidal attempt or completed suicide (e.g., 911 will be called).

Methods S4. MRI Quality Assurance

The following procedures are used to identify system problems that require technical attention, ensure strict adherence to study imaging protocols, guide the reacquisition of data during a scan session, and provide metrics of data quality that can be used to optimize subsequent analysis. In developing these procedures, the MCW Center for Imaging Research (CIR) team has relied on the rich experience of FBIRN (Functional Biomedical Informatics Research Network), a summary of which is available online, and used by the Human Connectome Project (HCP) (Keator et al., 2016; Marcus et al., 2013; Van Essen et al., 2012). These procedures are beyond the standard quality assurance program included as part of the American College of Radiology certification requirements which are implemented in the Daniel M. Soref Imaging Research facility to maintain clinical accreditation for neuro, spine, and other MRI modules.

The CIR operates two GE HealthCare Signa Premier 3.0T MRI systems, titled Soref01 and Soref02. Because software updates on the GE MRI platform have historically impacted the controlled timing parameters of longitudinal neuroimaging studies, the CIR has developed an extensive testing program for validating the stability of longitudinal neuroimaging studies during software updates. This program includes updating Soref01 and running software tests to ensure that key timing parameters do not change with neuroimaging protocols and, only after the performance is verified to match pre-update metrics or custom pulse sequence modifications are deployed to ensure that performance is matched, is Soref02 updated. When software updates are planned, thus, studies which have a high sensitivity to microsecond timing differences between sessions are encouraged to schedule exclusively on Soref02 during this validation process.

We use the FBIRN quality assurance (QA) procedure, based on echo-planar imaging (EPI) time series of a standard agar-filled phantom, for daily assessment of MRI system stability with each head RF coil that will be used that day. The purpose of the agar doping is to approximate the T1

and radiofrequency conductivity of brain tissue. Raw data from each QA scan are uploaded to an MCW server and analyzed using the functional MRI QA scripts developed by FBIRN. These tools provide a range of metrics, such as signal-to-noise-ratio and signal-to-fluctuation noise ratio (SFNR) of the images, the number of volumes with outliers (computed using Analysis of Functional NeuroImages [AFNI] 3dTout-count), the smoothness of the data (computed using AFNI 3dFWHMx), deviations in the volume center of mass, and noise spectral content. FBIRN has published criteria for acceptable values of each of these QA metrics.

All MRI data is uploaded to a central data server at MCW. Several indicators are obtained during image post-processing for monitoring data quality and protocol compliance. The EPI field of view is assessed visually to confirm complete coverage of the brain. Noise is assessed using maximal movement in each translation and rotation direction, SFNR, number of outlier volumes, deviations in the volume center of mass, and noise spectral content. Approximate signal loss in the EPI volume is calculated on a voxel-by-voxel basis from a whole-brain field map using the SIGLOSS tool in FSL.

Participants are trained in a mock scanner before their imaging sessions. Further, contoured padding is employed to physically ensure subject stability throughout the imaging session.

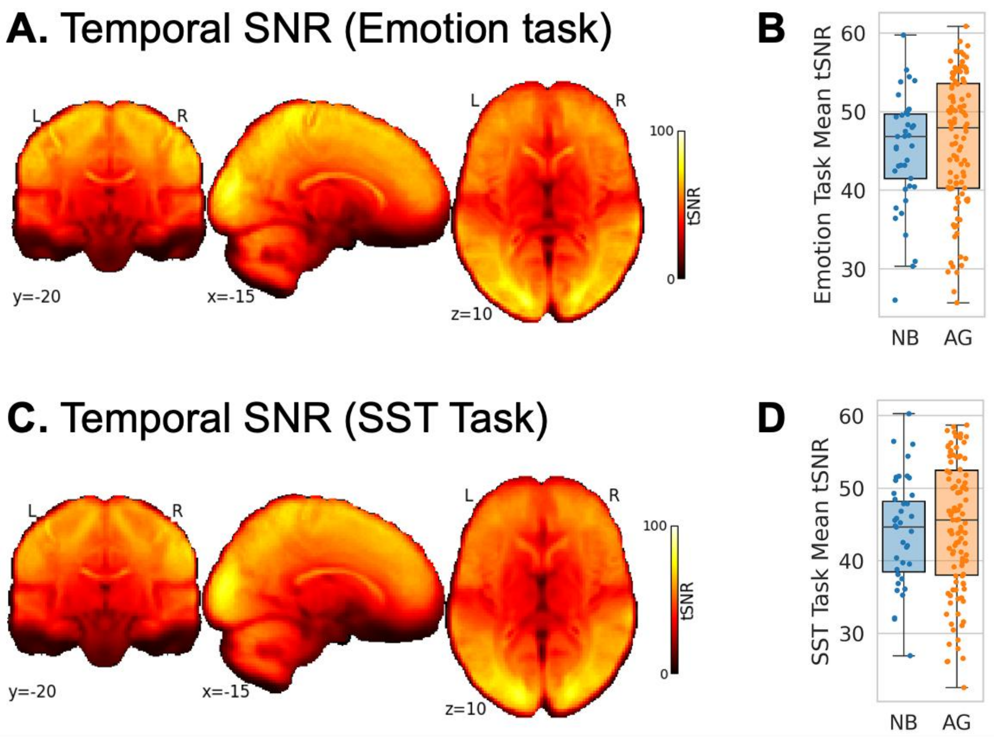

Figure S2. Summary of temporal signal-to-noise ratio (tSNR) from task fMRI

(A) Average temporal signal-to-noise ratio (tSNR) map from the emotion task before the motion and performance outliers were excluded (*n* = 141). (B) No significant group differences in the mean tSNR from the emotion task (*p* = 0.41; *n* = 141). (C) Average tSNR map from the SST task before the motion and performance outliers were excluded (*n* = 137). (D) No significant group differences in the mean tSNR from the SST task (*p* = 0.47; *n* = 137).

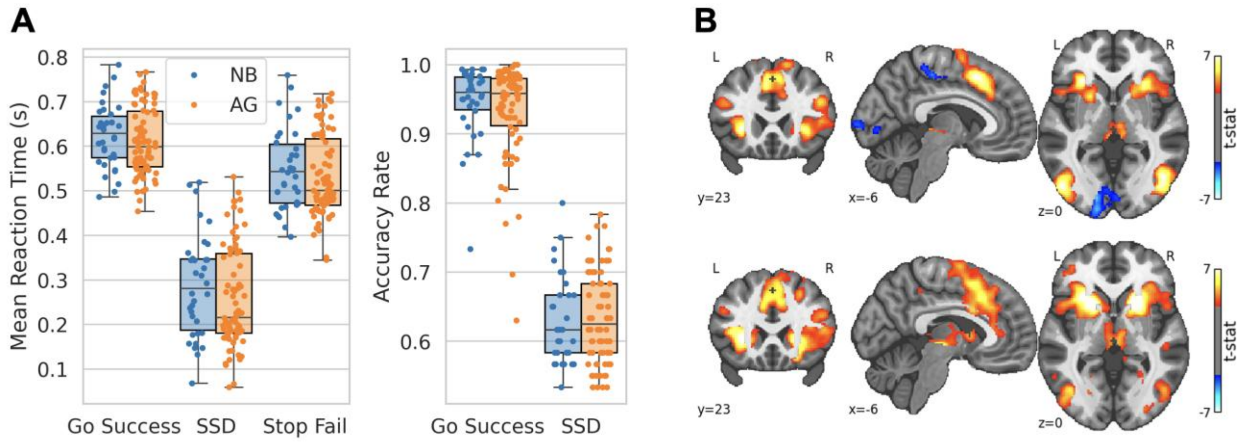

Figure S3. SST task activation using stringent performance flags

(A) Mean task response time and accuracy (*n* = 112, excluding motion outliers and performance outliers as proposed by Garavan et al. (2022)) (B) One-sample activation maps of successful stop minus correct go contrast (top) and unsuccessful stop minus correct go (bottom) (*n* = 112). Statistics were cluster corrected with a voxelwise $p < 0.001$ and cluster $\alpha = 0.05$.

References

Garavan, H., Chaarani, B., Hahn, S., Allgaier, N., Juliano, A., Yuan, D.K., Orr, C., Watts, R., Wager, T.D., Ruiz de Leon, O., Hagler, D.J., Jr., Potter, A., 2022. The ABCD stop signal data: Response to Bissett et al. Dev Cogn Neurosci 57, 101144.

Keator, D.B., van Erp, T.G.M., Turner, J.A., Glover, G.H., Mueller, B.A., Liu, T.T., Voyvodic, J.T., Rasmussen, J., Calhoun, V.D., Lee, H.J., Toga, A.W., McEwen, S., Ford, J.M., Mathalon, D.H., Diaz, M., O'Leary, D.S., Jeremy Bockholt, H., Gadde, S., Preda, A., Wible, C.G., Stern, H.S., Belger, A., McCarthy, G., Ozyurt, B., Potkin, S.G., 2016. The Function Biomedical Informatics Research Network Data Repository. Neuroimage 124, 1074-1079.

Marcus, D.S., Harms, M.P., Snyder, A.Z., Jenkinson, M., Wilson, J.A., Glasser, M.F., Barch, D.M., Archie, K.A., Burgess, G.C., Ramaratnam, M., Hodge, M., Horton, W., Herrick, R., Olsen, T., McKay, M., House, M., Hileman, M., Reid, E., Harwell, J., Coalson, T., Schindler, J., Elam, J.S., Curtiss, S.W., Van Essen, D.C., 2013. Human Connectome Project informatics: quality control, database services, and data visualization. Neuroimage 80, 202-219.

Van Essen, D.C., Ugurbil, K., Auerbach, E., Barch, D., Behrens, T.E., Bucholz, R., Chang, A., Chen, L., Corbetta, M., Curtiss, S.W., Della Penna, S., Feinberg, D., Glasser, M.F., Harel, N., Heath, A.C., Larson-Prior, L., Marcus, D., Michalareas, G., Moeller, S., Oostenveld, R., Petersen, S.E., Prior, F., Schlaggar, B.L., Smith, S.M., Snyder, A.Z., Xu, J., Yacoub, E., 2012. The Human Connectome Project: a data acquisition perspective. Neuroimage 62, 2222-2231.
